# Exploring comprehensive within-motif dependence of transcription factor binding in *Escherichia coli*

**DOI:** 10.1038/srep17021

**Published:** 2015-11-23

**Authors:** Chi Yang, Chuan-Hsiung Chang

**Affiliations:** 1 Institute of Biomedical Informatics, National Yang Ming University, Taipei, 11221, Taiwan; 2 Center for Systems and Synthetic Biology, National Yang Ming University, Taipei, 11221, Taiwan

## Abstract

Modeling the binding of transcription factors helps to decipher the control logic behind transcriptional regulatory networks. Position weight matrix is commonly used to describe a binding motif but assumes statistical independence between positions. Although current approaches take within-motif dependence into account for better predictive performance, these models usually rely on prior knowledge and incorporate simple positional dependence to describe binding motifs. The inability to take complex within-motif dependence into account may result in an incomplete representation of binding motifs. In this work, we applied association rule mining techniques and constructed models to explore within-motif dependence for transcription factors in *Escherichia coli*. Our models can reflect transcription factor-DNA recognition where the explored dependence correlates with the binding specificity. We also propose a graphical representation of the explored within-motif dependence to illustrate the final binding configurations. Understanding the binding configurations also enables us to fine-tune or design transcription factor binding sites, and we attempt to present the configurations through exploring within-motif dependence.

Most transcription factors (TFs) bind to specific DNA motifs to modulate gene expression. Understanding TF binding deciphers the control logic behind transcriptional regulatory networks, and modeling the binding motifs resolves the components of the networks. A TF binding model can be used to detect novel binding sites, describe the sequence requirements for TF-DNA recognition, and design binding sites for desired genetic regulations. Position weight matrix (PWM) is a classical way to model TF binding motifs in a position-independent manner^1^,^2^. Although PWM usually fits the binding motif well and reasonably approximates the true specificity of a TF^3^, it cannot capture within-motif dependence which has been described in previous structural, biochemical and statistical studies^4^,^5^,^6^,^7^. Modeling approaches that consider within-motif dependence can therefore obtain more accurate binding motif models.

There are currently two main modeling strategies that incorporate within-motif dependence to improve the predictive performance. In the first strategy, a binding model considers presumed dependencies: between adjacent positions^8^, among contiguous k-mers^9^, or between dinucleotides at any two positions^10^ in the DNA sequence. The work by Zhao *et al.* suggested that most of the within-motif dependence can be captured between adjacent positions^11^. The second strategy discovers within-motif dependence through searching correlated positions. Zhou *et al.* applied Gibbs motif sampling to search correlated position pairs from binding sequences^12^. Although the identified correlated pairs need not be neighbors, they have limited the pairs to be nonoverlapping. In a later study, Sharon *et al.* presented a feature motif model which couples motif discovery and feature selection for capturing within-motif dependence^13^. This approach searches correlated position pairs and allows overlaps among pairs. Although these modeling approaches assume within-motif dependence, they are either limited to some presumed arrangements without selection (strategy 1) or limited only to selected dinucleotides (strategy 2).

In addition to the two sequence feature-based strategies, an alternative approach is to include DNA shape-based features when describing a binding motif. These shape features can be derived from DNA sequences^14^ and incorporated into the binding motif modeling. These shape features encode the within-motif dependence implicitly to improve the performance of the model^15^.

In reality, we are far from obtaining the complete within-motif dependence of a TF binding. A TF may have base readout within more than one sub-region in a binding site, such as the previously resolved TF-DNA recognition structures of Ada^16^, HipB^17^ or PurR^18^ in *E. coli*. To model TF binding configuration for this type of interaction, both neighboring (within sub-region) and distant (between sub-regions) dependencies have to be considered. In addition, other dependencies may also exist for structural requirement such as the specific bending of DNA molecule for Fis binding^19^. Such dependencies cannot be directly retrieved by the aforementioned approaches. Therefore, we developed a strategy that can explore underlying within-motif dependence and present the binding configurations in TF-DNA recognition.

This study aims to improve the modeling of TF binding by searching within-motif dependence and construct robust and concise models. We first applied association rule mining techniques to explore potential dependencies. The resulting dependencies can consist of more than two distant positions that overcome the limitation of previous methods. We then used an elastic net regularized logistic regression model (ELRM), a machine learning approach, to describe the TF binding motif based on the dependencies. By employing feature selection, the ELRM can describe a binding motif more concisely. In addition, we also proposed an intuitive graphical representation of ELRM to illustrate the TF binding configurations. As a proof-of-concept demonstration, we analyzed the binding motifs of 86 TFs in *E. coli* K12 MG1655. The within-motif dependence discovered using our methodology can both explain the TF binding specificity and meet the TF-DNA recognition requirements. The core scripts of our proposed approach and the constructed ELRMs are publicly available at https://github.com/chiyang/ELRM.

## Results

### Exploration of within-motif dependences

**Figure 1:**
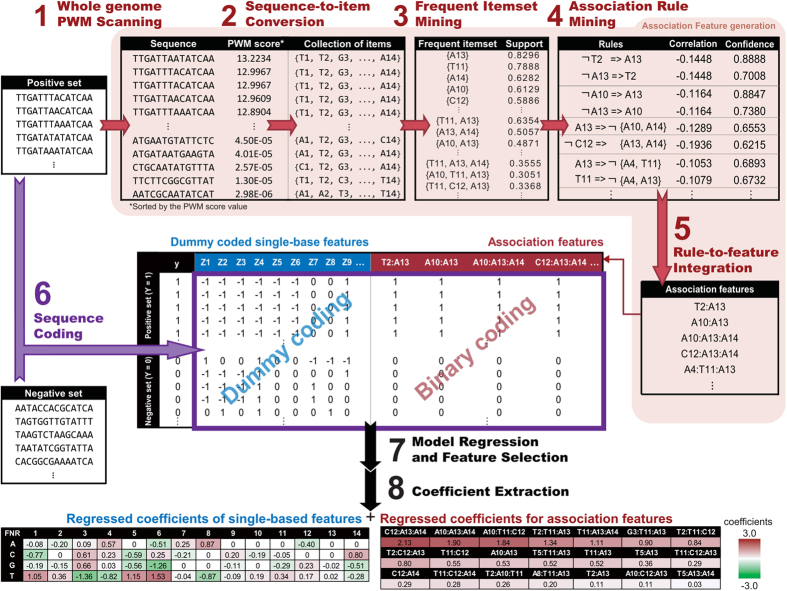
Flowchart for exploring within-motif dependence. Given a TF binding motif (composed of a collection of binding sequences used as the positive set), Steps 1 to 5 (red color) aimed to search for association features from similar sequences in a genome. In Step 6 (purple color), the training sequences were processed into coded single-base features through a dummy coding (see the coding table in Table 2). Step 6 also processed the training sequences to represent the state of association features with binary coding. Then, we applied an elastic net regularized logistic regression to construct the model (ELRM) and select relevant features simultaneously (Step 7). For interpreting the model, we extracted coefficients from the regressed model (Step 8). The regressed coefficients of coded single-base features can be decoded back to coefficients for the “non-coded” single-base features. The magnitude of the coefficients was represented by a color scale shown at the bottom right of this figure.

We introduce a novel approach to explore within-motif dependence of TF binding. (Fig. 1). In this study, two kinds of features were used in conjunction to describe a binding motif: (1) a single-base feature to describe a nucleotide base at a position and (2) an association feature to describe associations among single-base features. For example, a single-base feature “C2” describes the nucleotide base C at the second position in a motif, and an association feature “C2:G5” describes an association between C2 and G5. By considering a string of DNA sequence as a collection of single-base features, we first looked for potential association features in a binding motif (Steps 1 to 5 in Fig. 1). We then chose relevant features by constructing a model to describe the binding motif (Steps 6 to 8 in Fig. 1).

For every TF binding motif, we searched association features from the *E. coli* genome. Specifically, we constructed a PWM for each motif and performed a whole-genome PWM scanning. We then used the sequences that had PWM scores greater than zero to search for association features. After mining frequent itemsets and association rules on these sequences (Steps 3 and 4 in Fig. 1), we determined these single-base features in each association rule as potential dependencies. The association rules were integrated into association features as depicted in the Step 5 of Fig. 1. Then, we trained the elastic net regularized logistic regression model (ELRM) with these association features. The regularized regression allows us to select relevant single-base and association features by removing features of zero coefficients. We regard the remaining relevant association features as the within-motif dependence. The advantage of this approach is the capacity to accommodate comprehensive and complex within-motif dependence without presuming positional dependencies. The final ELRMs of the 86 *E. coli* TFs are summarized in Supplementary Table S1 and the full models are presented in Supplementary Table S2.

### TF binding specificity

To investigate whether the explored within-motif dependence can reflect TF binding specificity, we calculated the correlation between the ratio of association features in an ELRM and the width-normalized information content. We estimated the TF binding specificity using width-normalized information content as described in a previous study^20^. Among the 86 ELRMs, we observed a positive correlation of 0.73 between the ratio of association features and the information content (Supplementary Fig. S1). The positive correlation suggested that within-motif dependence can reflect TF binding specificity. Highly specific TFs tolerate little binding sequence variations, and thus have more dependencies to confine the binding pattern to one or a few conserved sequences. In contrast, low binding specificity TFs that allow many binding configurations will have few dependencies.

Conserved binding sequences are characteristics of highly specific TFs, yet small training sets may also produce binding sequences of limited variations. There are 25 ELRMs that contain only association features (Supplementary Fig. S1 and Supplementary Table S1). Among these, 18 ELRMs were trained from no more than six known binding sequences (Supplementary Table S1). The small training sets may result in overfitting and high ratio of association features. Therefore, TFs of high binding specificity are expected to have a high ratio of relevant association features in the constructed ELRMs, but not vice versa.

### Base readout in TF-DNA complexes

We further evaluated whether our explored within-motif dependence may reflect the TF-DNA recognition. In a TF-DNA complex, some side chains of a protein interact with nucleotide bases through hydrogen bonds, van der Waals interactions, or water-mediated interactions. The base readout usually plays roles in TF binding specificity. Based on the known TF-DNA binding profiles and the descriptions in structural studies (Table 1), around three-quarters of positions in base readout are involved with relevant association features (76.17% ± 18.55% (mean ± SD)). On the other hand, around 60% of the positions in association features are base readout positions. The base readout ratio of association features ranged from 20% to 100%. The large variation indicates that while the base readout contributed considerably to within-motif dependence in a few TFs, they only make up a part of the within-motif dependence in many TFs as indicated by the relatively low average (60%). In addition to base readout, the shape of a DNA molecule also contributes to TF-DNA recognition^21^. To investigate the roles of the within-motif dependence in the TF-DNA recognition, we presented examples in the following section.

### Participations of within-motif dependence in TF-DNA recognition

#### Between asymmetrical sub-regions

Ada is known to recognize the conserved A box (AAT, from positions 1 to 3) and B box (GCAA, from positions 10 to 13) of the promoters of the *ada* regulon by its N-terminal and C-terminal domains. The resolved X-ray crystal structure of N-Ada-DNA shows sequence-specific A box recognition while the NMR solution structure gives a better understanding of specific B box recognition^16^. The base readout around A and B boxes presented in Table 1 were based on the crystal and solution structures, respectively.

**Figure 2:**
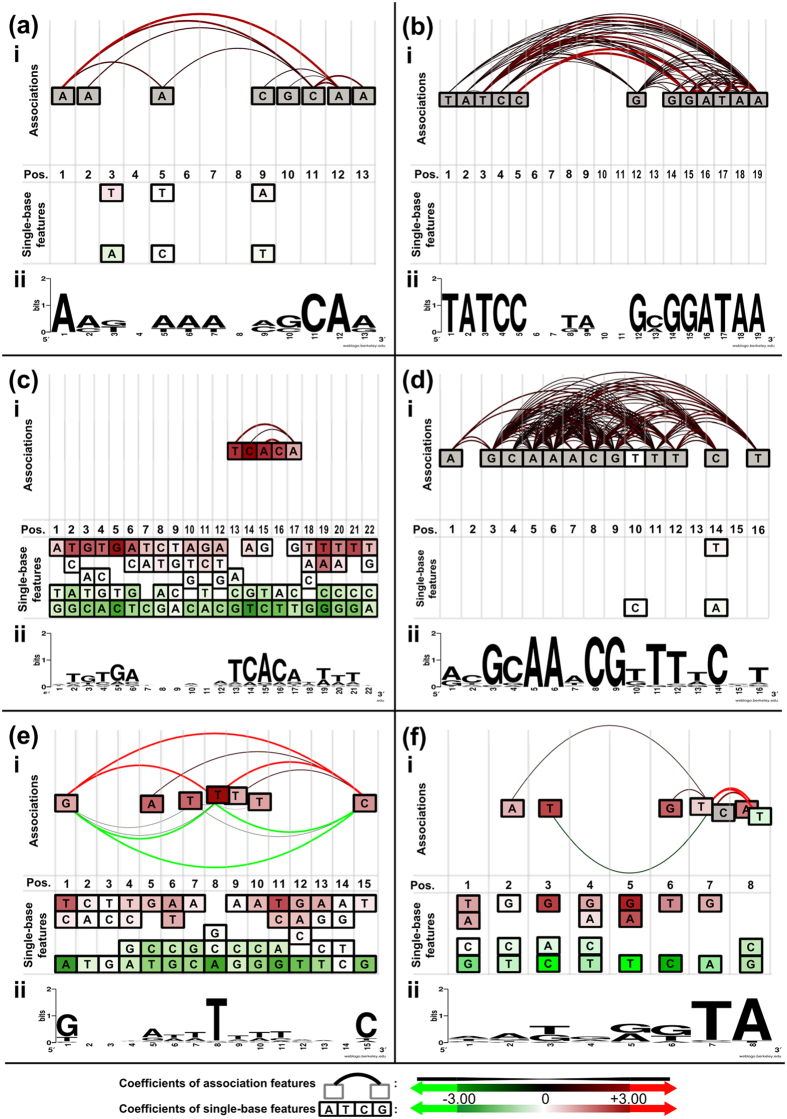
Graphical representations of constructed ELRMs. Sub-panel (i) presents our proposed graphical representation of ELRMs constructed for each of the six TFs: (**a**) Ada, (**b**) HipB, (**c**) CRP, (**d**) PurR, (**e**) Fis, and (**f**) NarL. Sub-panel (ii) shows the sequence logo representation of the original PWM of each TF. ELRM contains two types of features: single-base features and association features. Single-base features are depicted as rectangle boxes labeled with A/T/G/C, while association features are edges that link between single-base features (see Supplementary Methods for detailed explanations). The color of the boxes represents the magnitude of coefficients of the single-base features according to the color scale at the bottom of this figure. Both the width and color of the edges indicate the magnitude of the coefficients for association features, and the scale is also displayed at the bottom. For each sub-panel (i), single-base features which are part of the association features are shown at the top, whereas unassociated single-base features are displayed at the bottom.

Our model discriminates binding motifs by nine association and six single-base features (Fig. 2(a) and Supplementary Table S1). Six of the nine association features are involved with known base readout in Ada-DNA recognition, and they encompass the entire B box and the less-conserved C9. Our approach catches the essential features to reflect the Ada-DNA recognition despite the small positive training set.

#### Between repeated regions

Binding of dimeric TF complexes to DNA is a common regulatory event in a cell. The DNA motif consists of two unjoined repeated regions where each subunit of the homodimer recognizes a repeat. An example is the HipB protein that binds to DNA as a dimer (one on each strand)^17^. The HipB binding motif consists of inverted repeats of the consensus sequence TATCC. The two repeats are spaced by eight nucleotides. In the constructed ELRM of HipB, the model considered 25 association features and zero single-base features. Figure 2(b) illustrates extensive dependencies between the two repeated regions, and the model accurately reflects the binding characteristics of a HipB dimer.

In the HipB model, A19 is involved in 15 of the total 25 association features. The nineteenth position in the positive training sequences is always adenine, but guanine was used in the crystal structure^17^. Like the Ada example, only four positive training sequences were used to construct the HipB model. Replacing A19 with G19 greatly reduced the predicted probability from 0.9977 to 0.0198. The model became overfitted due to insufficient training sequences, much like the PWM of HipB described in a previous study^22^. Despite overfitting, A19 formed many valid associations with other positions based on the association rule mining but the significance remains to be resolved.

#### Within one of the two repeated regions

The cyclic AMP receptor protein (CRP) is a well-known global regulator and has the largest positive training set. Like HipB, CRP also recognizes a binding motif consisting of two inverted repeats. The repeated sequence is TGTGA, and two repeats are spaced by six nucleotides^23^. its model contains 83 single-base features and three association features (Supplementary Table S1). As a global regulator, CRP has a low binding specificity and can recognize a variety of sequence patterns. This property can be expressed by the high number of single-base features and few association features. As depicted in Fig. 2(c), the inverted repeats are expressed by the high coefficients of single-base features, whereas all of the three association features are located solely within the repeat on the right. This agrees with the fact that the right repeat is more conserved than the left repeat in the positive training set (Fig. 2(c)), and suggests that synergistic interactions may play roles in the CRP-DNA recognition.

#### For intensive base readout

The binding motif of PurR contains two inverted repeats as shown in Fig. 2(d), where each repeat is recognized by one subunit of the PurR homodimer^18^. From the resolved X-ray structure, DNA bound by PurR forms a kink structure at the central C8 and G9 nucleotides. In addition, A7 and its reverse complement T10 show severe unwinding. Extensive interactions between PurR and DNA are required for the energetic compensation of the unstacked base pairing to stabilize the kink structure^18^. The PurR-DNA recognition is also suggested to be dominated by base readout from an energy-based model^24^. Strong dependencies were expected to reflect this characteristic.

As expected, the ELRM of PurR had the highest number of association features in the 86 models. It contains 54 association features and four single-base features. Each association feature in the model constitutes of at least three single-base features (average 3.43 single-base features). This is the most complex among the 86 models (Supplementary Table S1). The graphical representation of the PurR ELRM in Fig. 2(d) depicts the complex association features. There are 87.04% (47/54) association features involved in connecting the two inverted repeats. As for the within-repeat associations, there are 68.52% (37/54) and 50% (27/54) association features involved connections within the left and right repeats respectively. PurR recognizes DNA through intensive base readout, and our approach expressed this characteristic with the high number of complex association features.

#### For structural requirements

Structural requirements can be captured by association features. As described in previous studies^15^,^21^, dependencies between adjacent positions can describe stacking interactions and short structural elements can be represented through continuous 3-mers. Here, we used Fis as an example to show association feature can be used to describe the underlying DNA shape. Fis is a well-known nucleoid-associated protein (NAP) in *E. coli*. Within the Fis-binding site, the most important base readout for each subunit of the Fis dimer in the Fis-DNA complex had been characterized by Stella *et al.*^19^. The G1 and C15 (guanine on the opposite strand) are base contacted with each of the Fis homodimer (see Table 1 for the base readout positions). In addition, the participation of the three central AT-rich base pairs (continuous 3-mers) in DNA bending was demonstrated in previous studies^19^,^25^. Replacement of the three AT-rich base pairs with GC-rich base pairs destabilizes the Fis-DNA complex. The DNA bending role of the central 3-mers is related to the minor groove widths and the stabilization of the complex^19^,^25^. Figure 2(e) depicts the relevant association features in the final model. The associations of G1, T8, and C15 are consistent with the result from the refined binding motifs, since the original motifs show higher information contents at these three positions (Fig. 2(e). The central three AT-rich positions are also part of the relevant association features, which suggests that the structural requirement for TF-DNA recognition can be expressed by the within-motif dependence.

#### For multiple binding configurations

In ELRM, the association features can also express multiple binding configurations. In the constructed model for NarL, the binding was summarized by 29 single-base features and six association features (Supplementary Table S1). As shown in Fig. 2(f), positions 7 and 8 each has two single-base features, i.e. T7/C7 and A8/T8. These single-base features form three association features, T7:A8, T7:T8, and C7:A8, and the summed coefficients are 6.80, 3.48, and 3.05, respectively. This case illustrates that the model allows multiple binding configurations where a position may have more than one single-base feature and form association with other positions.

There are 11 ELRMs that have multiple binding configurations, with at most two single-base features at a position. These multiple binding configurations occurred at one position in seven models; two positions in the models of MetJ and NarL; three positions in the NarP model; and four positions in the MalT model. Overall, these multiple binding configurations occurred at eighteen of the total 1380 positions of the 86 TF binding motifs. Thus, the data suggest that multiple-configuration model is rare.

Using the NarL model as an example, we found that taking association features into consideration can improve our understanding of the binding configurations. The consensus sequence of the NarL binding is AATGGGTA (Fig. 2(f)), and is also used in a structural study of the NarL-DNA complex^26^. Interestingly, our model generated a more optimal sequence, TAGGGGTA, that was not present in the positive training set. The two sequences differ only at the first and third positions. A native gel shift assay suggests that the TAGGGGTA is more favorable for the NarL binding than the AATGGGTA under the same binding condition^26^. Our model gives similar coefficients of the first three positions, AAT and TAG, but favors the TAG due to a negative coefficient of the association feature, T3:T7. The small negative coefficient thus differentiates the subtle differences between the two binding sequences.

## Discussion

Through constructing an ELRM, we interpreted the relevant association features as the within-motif dependence and used the dependence to describe TF binding motifs comprehensively. We used this approach to construct improved binding models for the 86 *E. coli* TFs. Without presumed dependencies in a binding motif, our within-motif dependence can express neighboring and distant dependencies composed of more than two positions. In addition, the explored within-motif dependence has a high correlation with the TF binding specificity and known base readout in the TF-DNA recognition. This suggests that the inclusion of our explored within-motif dependence in a model can improve our description of a binding motif.

During feature selection, the model can select relevant features from both coded single-base features and association features. Our approach reduced the number of selected features to almost half the amount used by the PWM (56.02 ± 22.96%, Supplementary Table S1). This means ELRM can use concise features to present a binding motif. Furthermore, the graphical representations can illustrate these features to facilitate our understanding of TF binding.

Although ELRM can be used as a binding site prediction tool (see Supplementary Methods for the workflow of sequence analysis with ELRM), we focused more on retrieving intact and concise TF binding characteristics. Our modeling approach sacrificed a little performance for the ability of feature selection. Considering fewer features can avoid overfitting to some extent and would leads to a reduced performance. The average cross-validation AUC of the 86 ELRMs is 0.9734 ± 0.0327 (mean ± SD). We consider this learning performance acceptable, although the reduced performance (1.7% ± 3%) is statistically significant compared to PWM, which has an average AUC of 0.9906 ± 0.0121 (Supplementary Table S3). Despite the slightly reduced performance, ELRM captures the majority of the binding characteristics of a TF.

This study applies a logistic regression model to learn and report a DNA sequence in a binary state of it being a TF binding site or not. The regression model can be adapted to make use of high-throughput experimental data. In addition, the TF-DNA binding may involve at least two mechanisms: through base readout and through the DNA shape in the binding site^21^. Although we showed ELRM can express the intense base readout or the structural requirement for TF binding, the DNA shape features may also be included as an additional feature to describe a binding motif^15^,^27^. In this way, we envision the model can directly present the TF-DNA recognition mainly through base readout, DNA shape features, or both. For engineering design purposes, we foresee the need for modeling detailed and wide-range TF binding configurations. Our ELRM is the first attempt to satisfy the need by exploring the comprehensive within-motif dependence.

## Methods

### TF binding motifs and *E. coli* genome sequence

We analyzed the binding motifs of 86 TFs in *E. coli* K12 MG1655 listed in Supplementary Table S1. These binding motifs were previously defined by Medina-Rivera *et al.*^22^. and were obtained from RegulonDB^28^. The genome sequence of *E. coli* K12 MG1655 was retrieved from NCBI RefSeq database (Accession No. NC_000913.2) and was used to search within-motif dependence.

### Whole genome PWM scanning

For each TF, we first constructed a PWM with a background model of the first-order Markov chain^1^. Then, the *E. coli* K12 MG1655 genome was scanned with the PWM (Step 1 of Fig. 1) using the MOODS tool^29^. Sequences were retained if their PWM scores were greater than zero. We considered each single-base feature in a sequence as an item, and the remaining unique sequences were converted into collections of items (Step 2 of Fig. 1).

### Frequent itemset mining

We applied the *Apriori* algorithm^30^ to find frequently co-occurring itemsets among the collections of single-base features. In this algorithm, a support of an itemset is defined as the proportion of the collections which contains the given itemset. The minimum support threshold was then used to partition the itemset into frequent and infrequent itemsets. Thus, a lower minimum support threshold will increase the amount of resulting frequent itemsets. With this step, we can find frequent sets of single-base features where the proportion of co-occurring itemsets is higher than the minimum support.

### Association rule mining

The association rule mining aims to find associations in the frequent single-base feature sets. For each frequent itemset X, we tested any two subsets A and B to see if a positive association *A* → *B* is valid. The two subsets must satisfy the conditions, *A* ∪ *B* = *X* and *A* ∩ *B* = 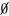. The two measures of an association rule *A* → *B*, confidence and correlation are, respectively, defined as^31^,^32^


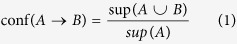



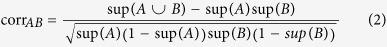


where sup(*A*) is the support value of A. The confidence, conf(*A* → *B*), describes a conditional probability *P*(*B*|*A*), while the correlation, corr_*AB*_, measures the correlation strength between sets A and B similar to a correlation coefficient. A positive association rule *A* → *B* is valid if conf(*A* → *B*) ≥ *mc* and |corr_*AB*_| ≥ *MCS*, where *mc* is the minimum confidence and *MCS* is the minimum correlation strength. In addition to the positive association rules, we also searched negative association rules *A* → ¬*B*, ¬*A* → *B*, and ¬*A* → ¬*B* (see Supplementary Method for detailed information). After the positive and negative association rule mining, a set of single-base features that forms the association rules was integrated as an association feature (Step 5 of Fig. 1).

### Model construction

In this study, a TF binding motif can be modeled through the logistic regression model as,


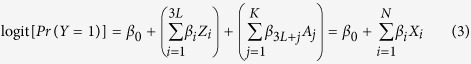


where *L* is the motif length, *K* is the number of association features, *Z*_*i*_ is the state of coded single-base feature generated by a dummy coding process, *A*_*j*_ is the state of the association feature, and *Y* represents the state of being a binding sequence. According to the dummy coding table (Table 2), each position has three coded single-base features in the regression model to represent the existence status of the four nucleotide bases. Therefore, a motif with the length *L* will have 3*L* coded single-base features for selection. As for association features, *A*_*j*_ is a binary code which was generated according to the state of each association feature present in a training sequence. Each training sequence was processed with this coding step before being inputted into the regression model (Step 6 of Fig. 1).

To prepare the data for model construction, the same refined sequences used in Step 1 (Fig. 1) were also used as the positive set in the training model. We generated the negative set by employing the following three procedures. First was to draw 10X sequences randomly from the scanning results of column-permuted PWMs as described in a previous study^22^. The threshold for the PWM scores was set at 0. Theoretically, results from a column-permuted PWM do not have the same characteristics as the original PWM unless the binding motif has low sequence complexity. Second was to generate 10X random sequences with the same overall GC percentage as sequences in the positive set. Third, 10X sequences were drawn from sequences with PWM scores less than 0 (calculated in Step 1). In this step, we set the lower bound of the scores to −2 to reduce computation time. In the end, a total of 30X non-redundant negative sequences and 1X positive sequences were obtained to train the regression model.

### Feature selection

When performing logistic regression, we applied an elastic net regularization^33^,^34^ to select relevant coded single-base and association features. This elastic net regularized logistic regression solves the minimization


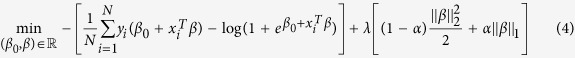


The elastic net regularization uses a mixture of ridge and lasso penalties. When *α* = 1, the regression is regularized by the lasso penalty, and the model tends to select one of the correlated features as a representative and discard the rest. This makes lasso a strong selection approach. When *α* = 0, the ridge regression is performed. The ridge penalty shrinks the regressed coefficients and tends to preserve all features without selection. We set *α* to be 0.5 so the resulting model preserves correlated groups of features without losing too many dependencies (see Supplementary Methods for detailed information). The *λ* in the minimization controls the degree of coefficient shrinkage in the regression model. This *λ* is a soft thresholding where an optimal *λ* can be obtained by a ten-fold cross-validation procedure. The optimal *λ* has minimum mean cross-validation error and the threshold for each constructed ELRM is given in Supplementary Table S1. The R package, glmnet^34^, was used to perform the elastic net regularized logistic regression.

After minimization, coefficients of coded single-base features were decoded to interpret TF binding configurations. Three coefficients of the coded single-base features (Table 2), *Z*_3(*p*−1)+1_, *Z*_3(*p*−1)+2_, and *Z*_3(*p*−1)+3_, can be further decoded for the four nucleotide bases as


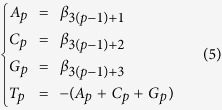


where *p* is the position in a motif. In this way, we can intuitively make use of the decoded coefficients of the single-base features to investigate the TF binding configuration. Features that have zero coefficients were considered as irrelevant and discarded. The remaining single-base features and association features were selected as relevant features to describe the binding motif.

### Cross-validation

N-fold cross-validation was applied to estimate the learning performance for each TF. Since the number of positive sequences varied from 4 to 236 for different TFs, a leave-one-out procedure was applied for TFs with ten or less positive sequences. The remaining TFs were assessed with a ten-fold cross-validation procedure. To summarize the learning performance, results from the N-folds were combined to produce a single estimate. Varied thresholds of the model responses were used to calculate the sensitivities and specificities, and an area under the curve (AUC) was used to assess the performance.

### Threshold settings

We screened 48 combinations of the three thresholds used in our approach to find the optimal settings for each TF (Supplementary Fig. S2). The tested threshold values were 0.1, 0.15, and 0.2 for the minimum support; 0.5, 0.6, 0.7, and 0.8 for the minimum confidence; 0.05, 0.1, 0.15, and 0.2 for the minimum correlation strength. We first searched for stable settings when (1) the 86 ELRMs have at most six cross-validation AUC outliers, and (2) the worst-case AUC is above 0.8. Then we searched for the optimal setting that gives the smallest standard deviation of cross-validation AUC among the stable settings. With this, we determined the optimal thresholds for minimum support, confidence and correlation strength to be 0.2, 0.6, and 0.1, respectively (Supplementary Fig. S2). The optimal setting also gives the maximum average AUC among all stable settings. Using the optimal setting, we constructed the ELRMs for the 86 *E. coli* TFs as shown in Supplementary Tables S1 and S2.

## Additional Information

**How to cite this article**: Yang, C. and Chang, C.-H. Exploring comprehensive within-motif dependence of transcription factor binding in *Escherichia coli*. *Sci. Rep.* **5**, 17021; doi: 10.1038/srep17021 (2015).

## Supplementary Material

Supplementary Information

## Figures and Tables

**Table 1:** Base readout positions in TF-DNA recognition.

TF	PDB id	Consensus sequence	No. of base readout in association features	No. of base readout	No. of positions in association features
Ada^16^	1zgw	******aA**TT******A**aag**CGCAA******	7	9	1
CRP^23^	1cgp	gtGtGAcatatg**TCaCa******ctttt	3	6	2
DnaA^35^	1j1v	tg**TTATcCACA******	8	8	2
FadR^36^	1h9t	atc******TGG****ta**C**ga**CC**A******ga	4	7	7
Fis^19^	3jr9	******G**ttTaaattttGag**C******	2	4	5
HipB^17^	3dnv	******TATCC**ccttaagg**GGATA******g	10	10	2
IHF^37^	1ihf	c**A**a****caaaT**TG******ata	3	4	3
LexA^38^	3jso	ta**CTG******T**A****tg**cgcaT**ACAG******ta	8	10	5
MarA^39^	1bl0	aT**TT******AGcAaaacG**TGG******Cat	5	11	3
MetJ^40^	1cma	a**GA**cg**T******C	3	4	3
MqsA^41^	3o9x	cctttt******AGGTT**Ata	5	6	5
NarL^26^	1je8	a**AT******gG**GTA******	5	6	0
PhoB^42^	1gxp	ct**G******Tcata******AA**gtt******G**Tc	4	6	6
PurR^18^	1pnr	ac**G******a******AAACGTTT**t**C******gt	10	10	3
PutA^43^	2rbf	c**GGTTGCA******Cc	6	8	1
Rob^44^	1d5y	aca**GC**a****ctgaatgtcaa	2	2	8

These base readout positions were collected based on the binding schema and descriptions in the corresponding studies as cited in the first column. Both strands of DNA sequence were used during mapping. For studies that provide half-site arrangement for symmetrical repeats, base readout positions of the other half were inferred. In the consensus sequence column, base readout positions are written in upper cases; associated positions are indicated by underlines; bold-face characters indicate both the associated positions and base readout positions.

**Table 2:** The dummy coding table.

	*Z*_*n*_	*Z*_*n*+1_	*Z*_*n*+2_
*A*_*p*_	1	0	0
*C*_*p*_	0	1	0
*G*_*p*_	0	0	1
*T*_*p*_	−1	−1	−1

To process the dummy coding, the single-base features of *A*_*p*_, *C*_*p*_, *G*_*p*_, and *T*_*p*_ were dummy coded to be *Z*_*n*_, *Z*_*n*+1_, and *Z*_*n*+2_ according to this table. The *p* is the position number in a motif and the *n* is defined as 3(*p *− 1) + 1.
