# Supplementary material for: Exploring comprehensive within-motif dependence of transcription factor binding in Escherichia coli
Source: Sci Rep. 2015 Nov 23;5:17021. doi: 10.1038/srep17021 (PMC4655474; doi:10.1038/srep17021)
Supplement: Supplementary Information [file srep17021-s1.pdf]

Supplementary Information

Exploring comprehensive within-motif dependence of transcription factor binding in *Escherichia coli*

Chi Yang[1] and Chuan-Hsiung Chang[1,2,*]

[1]Institute of Biomedical Informatics and [2]Center for Systems and Synthetic Biology, National Yang-Ming University, Taipei, Taiwan

*Correspondence to:

Dr. Chuan-Hsiung Chang
Center for Systems and Synthetic Biology
Institute of Biomedical Informatics
National Yang-Ming University,
No. 155, Sec. 2, Li-Nong St., Taipei 11221, Taiwan
Tel: 886-2-2826-7316
FAX: 886-2-2820-6754
E-mail: cchang@ym.edu.tw

Contents

Supplementary Methods.

Positive and negative association rule mining.

Selection of the α value during the elastic net regularization.

Workflow for sequence analysis with ELRM.

ELRM visualization.

Supplementary Figure S1. Correlations between information content and ratio of association features.

Supplementary Figure S2. Screening threshold settings.

Supplementary Figure S3. ELRM learning performance at different α values.

Supplementary Figure S4. The ratios of feature numbers at different α values.

Supplementary Figure S5. Graphical representation of ELRM.

Supplementary Table S1. Characterization of the 86 ELRMs

Supplementary Table S2. The resulting ELRMs for the 86 TFs.

Supplementary Table S3. Performance assessment of ELRM and other approaches.

References

Supplementary Method

Positive and negative association rule mining.

We considered each sequence as a collection of items. Each item is the single-base feature. For example, TTGAT, is a collection of five items, $\{T1, T2, G3, A4, T5\}$. We applied the *Apriori* algorithm[1] to search the frequent itemsets from collections. These collections were the non-redundant sequences which have PWM scores greater than zero. After performing the *Apriori* algorithm, we searched the frequent itemsets and their support values. For each frequent itemset X, we can find any two subsets A and B, which satisfy the conditions, $A \cup B = X$ and $A \cap B = \emptyset$. Then, we can calculate the support values for the negation itemsets ($\neg A \cup B$ or $\neg A \cup \neg B$) as follows:

$$\begin{aligned}
\sup(\neg A) &= 1 - \sup(A) \\
\sup(A \cup \neg B) &= \sup(A) - \sup(A \cup B) \\
\sup(\neg A \cup B) &= \sup(B) - \sup(A \cup B) \\
\sup(\neg A \cup \neg B) &= 1 - \sup(A) - \sup(B) + \sup(A \cup B)
\end{aligned}$$

Then, the confidence can be calculated by the following formulae.

$$\begin{aligned}
\mathrm{conf}(A \to B) &= \frac{\sup(A \cup B)}{\sup(A)} \\
\mathrm{conf}(A \to \neg B) &= \frac{\sup(A) - \sup(A \cup B)}{\sup(A)} = 1 - \mathrm{conf}(A \to B) \\
\mathrm{conf}(\neg A \to B) &= \frac{\sup(B) - \sup(A \cup B)}{\sup(\neg A)} = \frac{\sup(B) - \sup(A \cup B)}{1 - \sup(A)} \\
\mathrm{conf}(\neg A \to \neg B) &= \frac{1 - \sup(A) - \sup(B) + \sup(A \cup B)}{1 - \sup(A)} = 1 - \mathrm{conf}(\neg A \to B)
\end{aligned}$$

We can calculate the correlation for the rule $A \to B$ by the formula, $\mathrm{corr}_{AB} = \frac{\sup(A \cup B) - \sup(A)\sup(B)}{\sqrt{\sup(A)(1-\sup(A))\sup(B)(1-\sup(B))}}$, as the previous study.[2] We can easily replace the A or B with the negation forms, $\neg A and \neg B$. The valid positive and negative association rules can then be defined through the thresholds, the minimum confidence mc and minimum correlation strength, MCS, as the followed. The valid positive association rule is

$$A \to B, \text{ if } conf(A \to B) \geq mc, \text{ and } |corr_{AB}| \geq MCS,$$

and the valid negative association rules are

$$\begin{aligned}
A \to \neg B, &\text{ if } \mathrm{conf}(A \to \neg B) \geq mc, \text{ and } |\mathrm{corr}_{A\neg B}| \geq MCS \\
\neg A \to B, &\text{ if } \mathrm{conf}(\neg A \to B) \geq mc, \text{ and } |\mathrm{corr}_{\neg AB}| \geq MCS \\
\neg A \to \neg B, &\text{ if } \mathrm{conf}(\neg A \to \neg B) \geq mc, \text{ and } |\mathrm{corr}_{\neg A\neg B}| \geq MCS
\end{aligned}$$

After iterating all combinations of A and B for each itemset X, we can obtain valid positive and negative association rules.

Selection of the α value

There is no gold standard to set the α value during the elastic net regularization. Theoretically, the model tends to choose more features and have better data fitting at lower α (i.e. close to 0). In contrast, when setting a higher α (i.e. close to 1), the model tends to choose one or few features to represent each group of correlated features. Since our goal is to construct TF binding models with as few features as possible and also to provide a desired level of model interpretation, we want to preserve groups of correlated features if they help to explain the model response. In addition, instead of finding an optimal α for each TF binding model, we want to find a global setting of α for all TF binding model to reduce the exhausted parameter finding and accelerate the construction process. Therefore, setting the α to be 0.5 allows equal amount of ridge and lasso penalties. This α was considered as a balanced point between feature number and data fitting.

Empirically, we tested the learning performance at different α values. As the α grows, the overall variation of the learning performance was increased (Supplementary Fig. S3) and the selected feature numbers were reduced (Supplementary Fig. S4). These two figures present the continuously increased performance variation and reduced feature number. This agrees with the theoretical model behavior. Therefore, we set a global α at 0.5 in this study.

Workflow for sequence analysis with ELRM.

The Perl and R scripts used to construct ELRMs and perform sequence scan are publicly available at https://github.com/chiyang/elrm. Currently, the work was implemented for microorganisms with one chromosome. To perform genome scan or promoter analysis with ELRM, we suggest the following workflow.

1. Model construction
   (a) To construct an ELRM, fixed-length binding sequences of a TF are required as the positive sequences. These sequences can be obtained from Regulon DB, literatures, or ChIP-chip/ChIP-seq experiments.
   (b) Construct the PWM from the positive sequences.
   (c) Perform PWM scan on genome sequence(s) and collect sequences whose PWM scores are greater than zero. We suggest using PWM scanning tools which are suitable for chromosome-sized scanning. For exampe, MOODS,[3] TFM-scan,[4] or PWMscan (http://ccg.vital-it.ch/pwmtools/pwmscan.php) are suitable for genome-wide PWM scanning.
   (d) Perform *Apriori* algorithm for mining frequently co-occurring single-base features (Apriori.pl). In this step, the minimum support has to be specified.
   (e) Perform positive and negative association rule mining from the previous step and integrate rules into association features (FindRules.pl). In this step, the minimum confidence and the minimum correlation strength are required.
   (f) Code the sequences of training sets for model construction (SequenceCoding.pl). The training sets should contain positive and negative sequences. In this study, the negative sequences were generated as described in the main text. The training sequences of the 86 TFs in *E. coli* are also available from the ELRM website.
   (g) Regress the model (GetTheModel.R). After coding the training sets, an R script was implemented to construct the ELRM. An α value has to be given. The λ value is optional. When λ is not set, the script automatically performs 10-fold cross validation to generate a λ that gives the minimum mean cross-validation error. The output will include the λ value, and this λ can be given later to get the same model.
   (h) Visualize the ELRM. We provided an interactive user interface to investigate the model (please see the next section and Supplementary Fig. S5).
2. Sequence scan
   (a) In the testing steps, interested sequences such as promoter regions or the whole genome have to be coded into a matrix as shown in the Figure 1 in the main text. A Perl script was implemented to convert these long sequences into sliding windows of the motif length with 1-bp offset (SequenceCoding.pl).
   (b) After the sequence coding, an R script was provided to perform ELRM scan (ELRMscan.R).

For detailed usage of these scripts, please see the instructions on the ELRM website.

ELRM visualization

To assist in the understanding of the ELRM and fast investigation of the binding motif, we created a graphical representation of ELRM with an interactive user interface. The interface was written in HTML5 and can be easily viewed on web browsers that support HTML5. This interactive tool is also available on the ELRM website (https://github.com/chiyang/elrm). Supplementary Figure S5 explains the ELRM representation. Through this visualization, we aim to easily view the within-motif dependence and then interpret the characteristics of a binding motif. With the interactive user interface, one can inspect a single-base feature by clicking it and view its coefficient as well as the coefficients of the involved association features.

The user interface also allows users to test an interested sequence and see if the sequence is preferred for TF binding. The interface will highlight the involved single-base and association features of the given sequence and output a probability score of it being a binding site. Therefore, users can quickly test if their sequence of interest is a TF binding site and understand the reasons why the model describes the sequence as preferred/non-preferred.

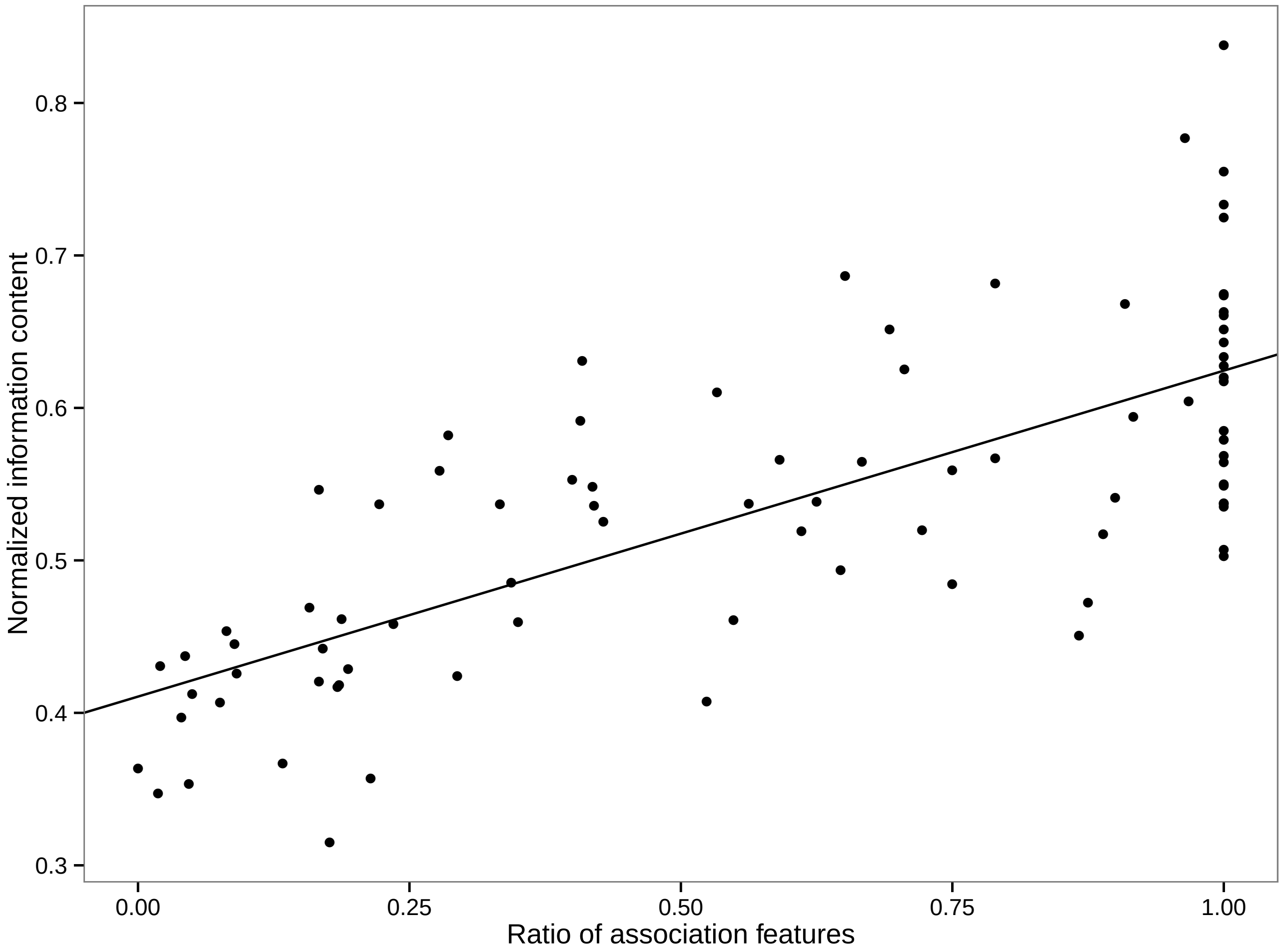

Supplementary Figure S1. Correlations between width-normalized information content and ratio of associations in ELRMs. This scatter plot shows the distribution of normalized information contents versus the ratio of association features. The ratio of association features is the number of association features over the total feature number (i.e. total number of coded single-base features and association features). The linear regression line shows the Pearson correlation coefficient of 0.71.

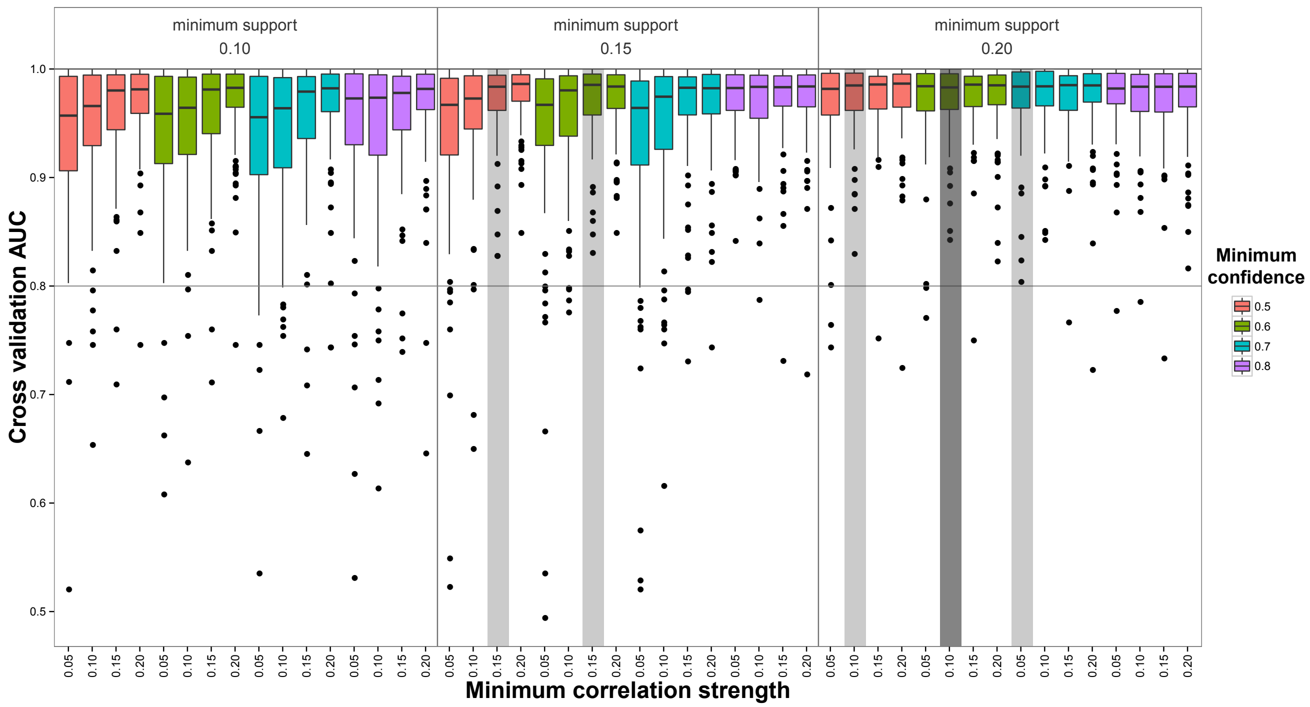

Supplementary Figure S2. Screening threshold settings. Three thresholds (minimum support, minimum confidence, and minimal correlation strength) determine the quantity and quality of association features for our model construction. We constructed ELRMs with the 48 combinations of three minimum supports (0.1, 0.15, and 0.2), four minimum confidences (0.5, 0.6, 0.7, 0.8), and four minimum correlation strengths (0.05, 0.1, 0.15, 0.2). For each threshold set, the 86 TF binding models were trained and the cross-validation AUCs of these 86 models were used to assess the learning performance and shown in the boxplot. The gray bars indicate the threshold sets which passed our criteria, i.e. the worst cross-validation AUC is more than 0.8 and the number of outliers is less than seven. The dark gray bar shows the threshold set which passed the criteria with the smallest standard deviation.

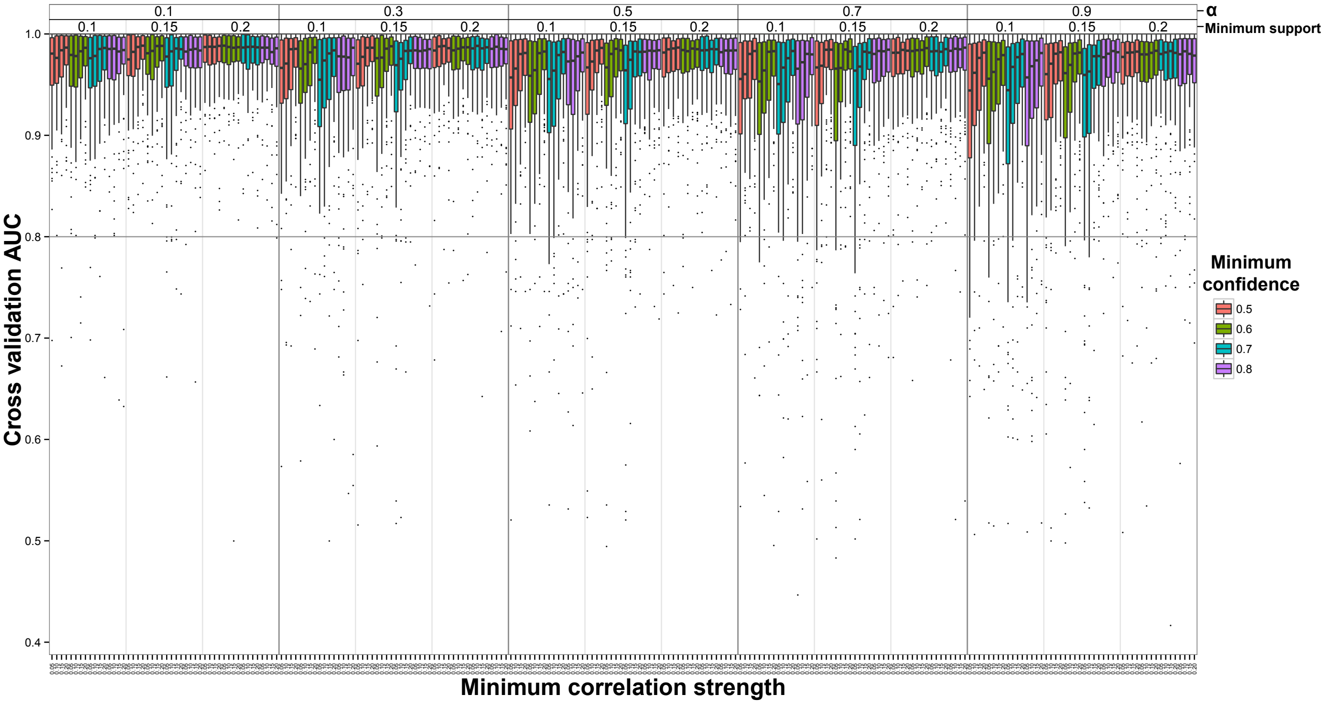

Supplementary Figure S3. ELRM Learning performance at different α values. This figure is an extension of the supplementary Fig. S2. In addition to the 48 combinations of parameters at the $\alpha = 0.5$, we tested additional α values: 0.1, 0.3, 0.7, and 0.9. The figure shows the overall variation increases with larger α. The number of outliers also increased at higher α.

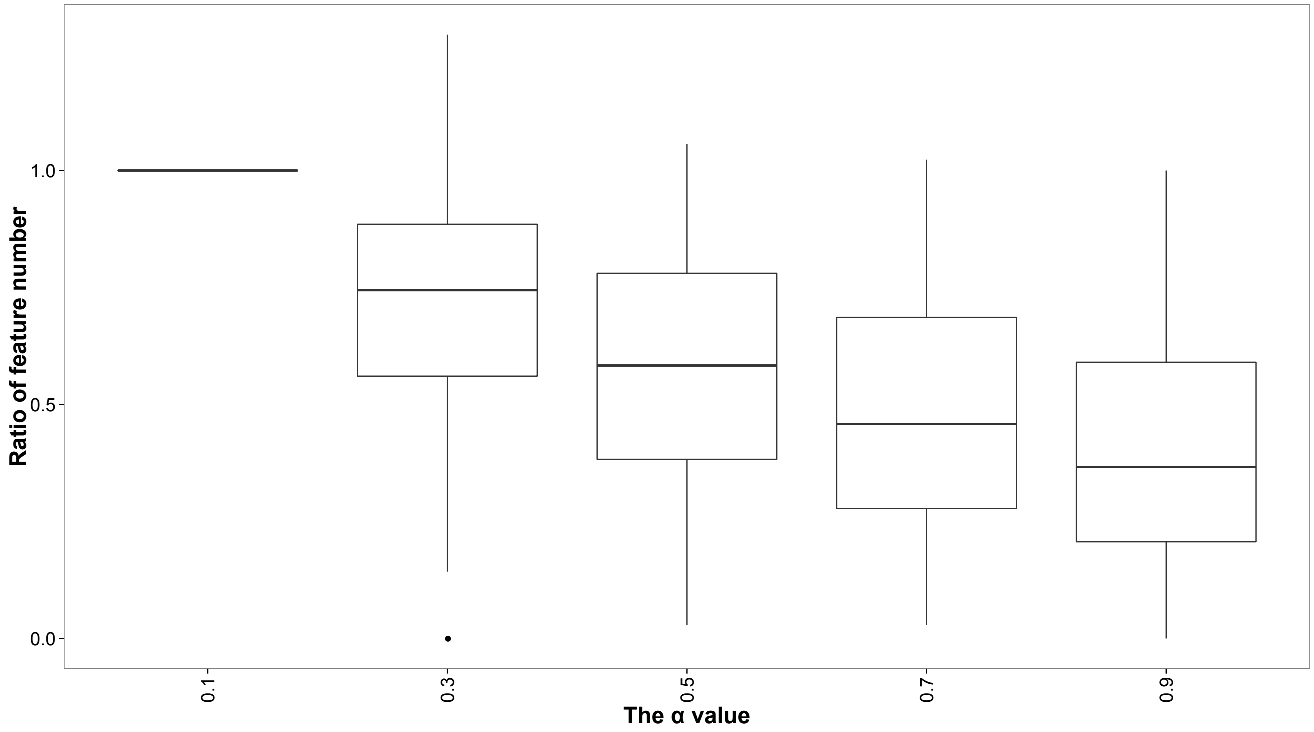

Supplementary Figure S4. The ratios of feature numbers at different α values. The ratios were calculated relative to the feature number at the α of 0.1 under the same parameter settings. For each α, models from the 48 configurations were constructed, and the feature numbers were summarized and presented in this boxplot.

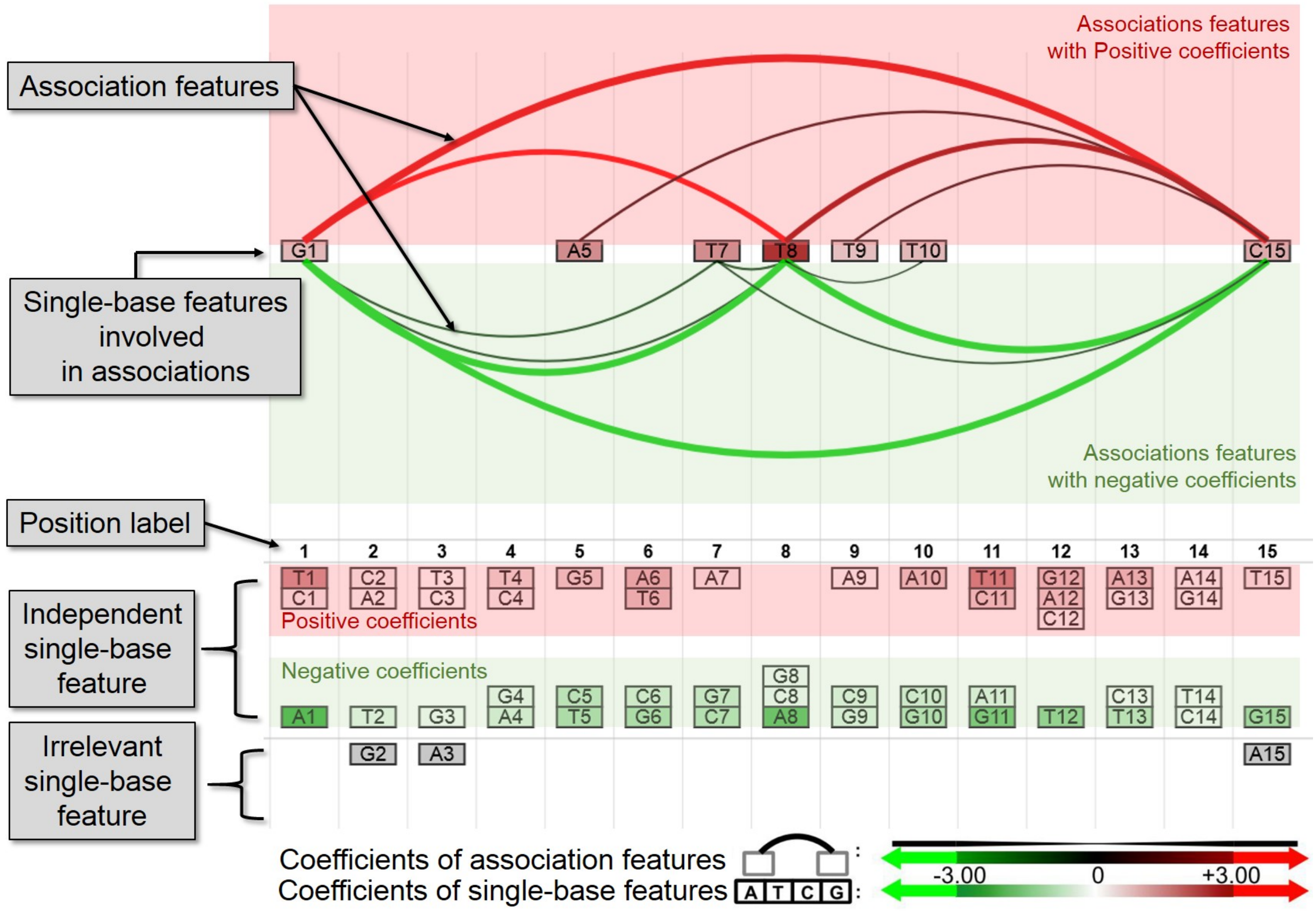

Supplementary Figure S5. Graphical representation of ELRM. The representation contains three parts, association features, independent single-base features, and irrelevant features. The single-base features were represented as rectangular boxes, and association features were edges that link among the boxes. It is worth noting that an association feature may be constituted of more than two single-base features. In this case, any two single-base features in the association feature will be joined by an edge. For example, in the above figure, the three association features G1:T8, G1:C15, and T8:C15 had positive coefficients and G1:T8:C15 had a negative coefficient. Therefore, the three single-base features G1, T8, C15 were linked by both red (positive) and green (negative) edges. On the other hand, two single-base features may be involved in more than one association features. This explains the presence of multiple edges that link between two single-base features. Although this ELRM representation can not directly present which single-base features form an association feature, we created a web-based interactive user interface that provides tooltips to assist investigation of the association features (see Supplementary Methods).

Supplementary Table S1. Characterization of the ELRMs for the 86 TFs. This table presents summarized information about the constructed ELRMs. The motif length, positive training set size, and the normalized information content are related to the original motif characteristics. The complexity value is the average number of single-base features in an association feature. The feature reduction was calculated by comparing to the PWM approach, which uses the feature number of four times the motif length.

TF	Motif length	Positive training set size	Normalized information content	No. of coded single-base featues	No. of association features	Complexity	Feature reduction (%)
Ada	13	4	0.4843	3	9	2.00	76.92
AgaR	19	11	0.6516	0	25	3.08	67.11
AraC	19	15	0.3970	48	2	2.00	34.21
ArcA	15	75	0.4256	40	4	2.00	26.67
ArgP	18	16	0.3636	39	0	0.00	45.83
ArgR	18	27	0.5368	26	13	2.54	45.83
AscG	13	7	0.5374	0	13	3.00	75.00
AsnC	12	4	0.5849	0	9	2.56	81.25
BaeR	17	4	0.5790	0	14	2.50	79.41
CRP	22	236	0.3535	61	3	2.00	27.27
CaiF	15	4	0.7247	0	29	3.00	51.67
CpxR	15	57	0.4181	44	10	2.40	10.00
Cra	18	32	0.5483	25	18	2.78	40.28
CsgD	11	21	0.4286	25	6	2.00	29.55
CysB	37	10	0.3470	53	1	2.00	63.51
CytR	18	17	0.4074	10	11	2.64	70.83
Dan	7	5	0.6681	1	10	2.80	60.71
DcuR	17	6	0.5647	6	12	2.58	73.53
DeoR	11	7	0.5371	0	9	2.56	79.55
DgsA	23	7	0.6277	0	19	2.95	79.35
DnaA	11	11	0.7550	0	15	2.93	65.91
EvgA	17	7	0.7333	0	32	3.00	52.94
ExuR	17	6	0.6335	0	19	3.00	72.06
FNR	14	83	0.5359	29	21	2.71	10.71
FadR	16	14	0.5669	4	15	2.67	70.31
FhlA	14	7	0.4937	6	11	2.36	69.64
Fis	15	211	0.3150	42	9	2.22	15.00
FlhDC	16	16	0.4691	32	6	2.33	40.63
Fur	18	47	0.5820	35	14	2.64	31.94
GadE	18	5	0.5199	5	13	2.62	75.00
GadW	20	17	0.4308	48	1	2.00	38.75
GadX	19	24	0.3669	39	6	2.00	40.79
GalR	16	12	0.5659	9	13	2.85	65.63
GalS	15	12	0.6253	10	24	2.92	43.33
GcvA	24	4	0.4594	13	7	2.29	79.17
GlpR	20	17	0.4537	45	4	2.00	38.75
GntR	18	9	0.5411	1	9	2.67	86.11
HNS	13	48	0.4123	38	2	2.00	23.08
HipB	19	4	0.6173	0	25	3.00	67.11
IHF	13	95	0.4420	39	8	2.50	9.62
IclR	7	10	0.6747	0	8	2.50	71.43
IscR	22	11	0.4066	49	4	2.00	39.77
LeuO	18	4	0.5352	0	16	2.88	77.78
LexA	20	37	0.6042	1	30	3.37	61.25
Lrp	12	72	0.3569	33	9	2.00	12.50
MalT	10	15	0.6816	4	15	2.60	52.50
MarA	19	22	0.4206	40	8	2.50	36.84
MelR	18	5	0.5489	0	10	3.00	86.11
MetJ	7	22	0.6515	4	9	2.22	53.57
MetR	15	5	0.5170	1	8	2.63	85.00
MlrA	30	4	0.4615	13	3	2.67	86.67

TF	Motif length	Positive training set size	Normalized information content	No. of coded single-base featues	No. of association features	Complexity	Feature reduction (%)
MntR	21	6	0.6605	0	24	2.92	71.43
ModE	24	7	0.4724	2	14	2.93	83.33
MqsA	14	5	0.5254	12	9	2.00	62.50
MqsAMqsR	19	4	0.5027	0	11	2.91	85.53
Nac	15	12	0.4451	41	4	2.25	25.00
NagC	22	17	0.5529	15	10	2.80	71.59
NanR	5	6	0.8379	0	6	3.00	70.00
NarL	8	91	0.5369	21	6	2.00	15.63
NarP	8	20	0.5914	16	11	2.18	15.63
NhaR	15	6	0.5373	7	9	2.44	73.33
NrdR	17	6	0.5642	0	15	3.00	77.94
NsrR	11	39	0.6866	15	28	2.89	2.27
NtrC	17	17	0.5586	26	10	2.80	47.06
OmpR	19	20	0.4371	44	2	2.00	39.47
OxyR	17	32	0.4168	40	9	2.22	27.94
PdhR	16	10	0.6101	7	8	2.25	76.56
PhoB	16	19	0.4853	21	11	2.27	50.00
PhoP	17	32	0.4609	14	17	2.82	54.41
PurR	16	21	0.7769	2	54	3.43	12.50
PutA	10	5	0.6736	0	16	3.00	60.00
RcsAB	15	6	0.5464	15	3	2.67	70.00
RcsB	14	7	0.5385	6	10	2.30	71.43
RelBRelE	12	4	0.5071	0	8	2.13	83.33
RhaS	17	6	0.4241	12	5	2.00	75.00
Rob	17	10	0.4507	2	13	2.46	77.94
RstA	14	4	0.6629	0	21	2.86	62.50
RutR	16	5	0.5499	0	10	2.50	84.38
SlyA	11	6	0.5942	1	11	2.18	72.73
SoxS	18	24	0.4581	26	8	2.75	52.78
TorR	9	8	0.6307	13	9	2.22	38.89
TrpR	16	6	0.6431	0	16	2.75	75.00
TyrR	17	19	0.5591	8	24	3.00	52.94
UlaR	17	4	0.5687	0	28	2.86	58.82
UxuR	15	4	0.6200	0	18	2.94	70.00
XylR	16	4	0.5192	7	11	2.27	71.88

Supplementary Table S2. The resulting ELRMs for the 86 TFs. This table shows the resulting ELRMs trained from annotated binding motifs of the 86 TFs in *E. coli*. The λ is given as 18 decimal digits of precision to reproduce the same model.

TF	The ELRM	The optimal λ
Ada	$\text{logit}[Pr(Y=1)] = -7.7230 - 0.2906[Z7] - 0.0668[Z14] + 0.1499[Z25] + 2.4925[A1:A12] + 1.9199[A1:C11] + 1.6965[C11:A13] + 1.6068[C11:A12] + 1.2099[A1:A5] + 0.8827[A2:C11] + 0.7258[A5:C11] + 0.6063[C9:A12] + 0.2340[G10:A12]$	0.007595306010045220
AgaR	$\text{logit}[Pr(Y=1)] = -7.6367 + 2.3345[T3:T8:T12:T13] + 1.7853[T3:T8:T9:T12] + 1.0426[T3:T4:T12:T13] + 0.9788[C5:T8:T13] + 0.8230[T3:T13:T18] + 0.6477[T3:C5:T9] + 0.6357[T4:T8:T14] + 0.4626[T3:C5:T12] + 0.4289[T8:T13:T18] + 0.3784[T9:T12:T13] + 0.3677[T4:T8:T18] + 0.3394[C1:T8:T13] + 0.3275[T3:C5:T8] + 0.2895[C1:T8:T12] + 0.2881[T4:T13:T18] + 0.2663[T3:C5:T13] + 0.2570[C1:T2] + 0.2020[T3:T8:T18] + 0.1772[T2:T8:T12] + 0.1671[T4:T9:T14] + 0.1366[T8:T12:T18] + 0.1256[C1:T3:T12] + 0.1205[T2:T8:T13] + 0.0267[C1:T3:T13] + 0.0030[C1:T3:T8]$	0.007054428912606220
AraC	$\text{logit}[Pr(Y=1)] = -15.6873 - 1.5980[Z1] - 1.4751[Z3] + 2.4190[Z4] + 0.9836[Z6] - 0.9834[Z7] - 1.3006[Z8] + 0.1043[Z9] + 0.2456[Z10] - 1.0955[Z11] + 1.3421[Z12] - 0.7215[Z13] - 0.8515[Z14] + 1.1254[Z15] + 1.5282[Z16] + 0.0970[Z17] - 0.1984[Z18] - 0.8731[Z19] + 2.3072[Z20] - 1.0236[Z21] - 0.2323[Z22] - 1.2644[Z23] - 0.1284[Z24] + 1.3306[Z25] - 1.7168[Z27] - 0.1373[Z28] + 1.1768[Z29] - 1.4441[Z30] - 1.7313[Z31] + 1.9377[Z32] - 1.0423[Z33] - 0.0594[Z34] - 1.4063[Z36] - 0.4850[Z37] - 0.5590[Z38] - 0.2708[Z41] + 0.5399[Z42] - 0.4746[Z43] + 1.6792[Z44] - 0.0310[Z45] - 0.9195[Z46] - 0.9448[Z47] + 0.2422[Z49] + 1.3385[Z50] - 0.1139[Z52] + 0.5206[Z53] - 1.5148[Z54] - 0.4053[Z55] - 1.6319[Z56] + 2.4789[C15:T16] + 0.0226[T3:C15]$	0.000225191042726985
ArcA	$\text{logit}[Pr(Y=1)] = -11.4029 + 0.5705[Z1] - 0.3683[Z2] - 0.6699[Z3] + 1.3182[Z4] - 0.7949[Z5] - 0.7434[Z6] - 0.4009[Z7] + 1.0018[Z8] + 0.5108[Z10] - 0.5513[Z11] + 0.2533[Z13] + 0.2582[Z14] - 1.1696[Z15] + 0.3823[Z16] - 1.4226[Z17] + 0.6844[Z18] + 0.1040[Z20] - 2.1676[Z21] - 0.3451[Z22] + 0.6585[Z23] + 0.0118[Z24] + 0.9938[Z25] - 1.7168[Z26] + 1.4596[Z27] + 0.0443[Z28] - 0.8505[Z29] - 0.7366[Z30] - 0.1886[Z33] + 1.9191[Z34] - 1.0742[Z35] - 1.2538[Z36] + 0.4318[Z37] + 0.8602[Z38] - 1.0292[Z39] + 0.8415[Z40] + 1.7834[Z41] - 1.4035[Z42] + 0.8980[Z43] + 0.7105[Z44] - 1.1294[Z45] + 1.7731[T11:A12] + 1.4856[A13:C14] + 0.8914[T10:A13] + 0.0806[A13:A14]$	0.000592643985327342
ArgP	$\text{logit}[Pr(Y=1)] = -12.0616 + 0.5795[Z1] + 1.7634[Z2] - 0.4237[Z5] - 1.4455[Z6] - 1.4046[Z8] - 0.1846[Z9] + 0.9882[Z10] - 1.4287[Z12] - 0.0580[Z14] - 0.8796[Z15] - 0.5588[Z16] - 1.2776[Z17] - 1.0222[Z20] + 1.3808[Z21] - 0.9362[Z22] - 1.2857[Z24] - 0.7368[Z25] + 1.1398[Z26] + 1.4837[Z27] + 0.2276[Z28] - 0.0316[Z29] + 0.6647[Z31] + 1.4946[Z32] + 0.0807[Z34] - 1.4775[Z35] - 1.4363[Z37] - 0.9330[Z38] + 0.2086[Z39] + 1.2188[Z40] - 1.3917[Z41] + 0.9448[Z42] - 0.0118[Z44] - 0.1710[Z45] + 0.1712[Z46] - 0.4684[Z48] + 0.0344[Z49] - 0.7416[Z50] + 0.2978[Z51] + 0.6776[Z53]$	0.001186884310037420
ArgR	$\text{logit}[Pr(Y=1)] = -10.4893 - 0.3349[Z2] - 0.8364[Z6] + 0.4155[Z7] - 0.0908[Z11] - 0.1826[Z13] - 0.2914[Z14] - 0.1156[Z15] - 0.3002[Z16] + 0.9274[Z18] + 0.7607[Z19] + 0.3982[Z22] + 0.2670[Z24] - 0.5287[Z27] - 0.6349[Z29] - 0.0408[Z32] - 0.5872[Z33] - 0.3829[Z35] - 0.1802[Z38] - 1.3600[Z39] - 0.5308[Z43] - 0.1987[Z44] - 0.9323[Z45] + 0.8531[Z48] + 0.0858[Z50] + 0.2357[Z52] + 0.5265[Z54] + 2.1645[A8:C17] + 1.7591[T9:A14:A18] + 1.4662[T12:C17:A18] + 1.2139[A14:C17:A18] + 1.0983[T12:A14:A18] + 0.9823[T10:A14] + 0.9325[A3:A14:A18] + 0.6936[A13:A14] + 0.6770[G6:A18] + 0.5124[T9:T12:A18] + 0.4106[A14:C17] + 0.3725[A8:A14:A18] + 0.2957[T13:A14]$	0.002049652633270570
AscG	$\text{logit}[Pr(Y=1)] = -10.4915 + 3.2347[G6:A8:C10] + 2.7370[G6:A9:C10] + 2.0512[T5:G6:A8] + 2.0333[T5:G6:C10] + 1.8785[T5:A9:C10] + 1.3203[T5:A8:C10] + 1.2506[T5:G6:A9] + 0.7725[G6:A7:A8] + 0.6036[A8:A9:C10] + 0.4550[G6:A7:A9] + 0.3096[G6:A8:A9] + 0.2146[T5:A8:A9] + 0.1096[T5:G6:A7]$	0.000382373516890067
AsnC	$\text{logit}[Pr(Y=1)] = -8.0123 + 3.9864[T4:T5:G9] + 2.9059[T5:T8:G9] + 2.8189[T4:T8:G9] + 0.8377[T4:T5:T8] + 0.4615[T4:T5:T12] + 0.3714[T8:T12] + 0.1992[T4:G6] + 0.0429[T8:G9] + 0.0270[T3:G9]$	0.003971612614051720
BaeR	$\text{logit}[Pr(Y=1)] = -7.1399 + 1.8906[T1:T2:C3] + 1.6775[C3:T4] + 1.4507[T1:A10:T11] + 1.2211[T2:C6:T11] + 0.6696[T2:A10:T11] + 0.6281[T1:T2:T11] + 0.5546[A10:T12] + 0.4754[T11:G13] + 0.4728[T2:C6:A10] + 0.2353[G16:C17] + 0.2110[T1:T2:A10] + 0.1540[T4:C6] + 0.1105[T2:C3] + 0.0960[C6:T8]$	0.011748074024848300
CRP	$\text{logit}[Pr(Y=1)] = -14.6532 + 0.4745[Z1] - 0.4267[Z3] - 0.7640[Z4] + 0.0539[Z5] - 0.8372[Z6] - 0.1252[Z7] - 1.1262[Z8] + 1.4700[Z9] - 1.1077[Z10] - 0.0692[Z11] - 0.1709[Z12] - 2.1165[Z14] + 2.2567[Z15] + 1.4322[Z16] + 0.0970[Z17] - 0.6395[Z18] + 0.1406[Z19] - 0.7395[Z20] - 0.2971[Z22] + 0.4228[Z23] - 0.3809[Z24] - 0.1815[Z25] - 0.0520[Z26] + 0.0154[Z27] + 0.6585[Z28] - 0.8621[Z29] + 0.0241[Z30] - 0.6650[Z31] + 0.3887[Z32] + 0.5148[Z33] + 0.7238[Z34] - 0.9344[Z35] + 0.0152[Z36] - 0.1602[Z37] - 0.4221[Z38] - 1.2900[Z39] + 0.3667[Z40] + 2.5464[Z41] - 0.3415[Z42] + 1.9398[Z43] - 1.4374[Z44] + 0.0511[Z45] - 0.1359[Z46] + 1.6104[Z47] + 0.7660[Z49] - 0.2374[Z50] + 0.0746[Z51] + 0.2113[Z52] + 0.0595[Z53] - 0.9537[Z54] + 1.2390[Z55] - 0.8030[Z56] - 2.2399[Z57] + 0.2940[Z58] - 0.4597[Z59] - 0.9867[Z60] - 0.3296[Z62] - 1.3277[Z63] - 0.4048[Z64] - 0.2664[Z65] + 0.0249[Z66] + 2.0506[A15:C16] + 1.7861[T13:A17] + 0.5810[C14:A17]$	0.000254061904572609
CaiF	$\text{logit}[Pr(Y=1)] = -8.1171 + 1.5620[A1:T6:G10] + 1.5551[A5:A7:A11] + 1.2934[A7:G10:A11] + 0.8586[A1:C3:A11] + 0.7095[C3:T6:A13] + 0.6797[C3:A11:A13] + 0.5709[A4:T6:G10] + 0.4720[A1:C3:T9] + 0.4690[A1:T8:A11] + 0.4422[A4:T9:A11] + 0.4333[C3:T9:A11] + 0.4171[A1:A4:A12] + 0.3906[C3:A12:A13] + 0.3289[T6:T8:A11] + 0.2326[C3:T8:A11] + 0.2220[A4:A7:A11] + 0.1986[T6:A7:A11] + 0.1778[G10:A11:A13] + 0.1740[A4:A7:A12] + 0.1733[T9:G10:A13] + 0.1686[A1:A5:A12] + 0.1602[A1:T6:A7] + 0.1447[A1:A7:T8] + 0.1436[T6:T9:A13] + 0.1382[A1:T6:A13] + 0.1227[A1:C3:A5] + 0.1198[C3:A7:A11] + 0.0884[T8:A11:A12] + 0.0091[A1:G10:A11]$	0.003147435075522700
CpxR	$\text{logit}[Pr(Y=1)] = -11.1970 - 1.8046[Z1] + 0.4196[Z2] + 0.7315[Z3] - 1.0290[Z4] - 0.3410[Z5] + 0.3117[Z6] + 0.5124[Z7] + 0.1651[Z8] - 0.7339[Z9] + 1.4933[Z10] - 1.0464[Z11] - 1.0715[Z12] + 1.1575[Z13] - 0.0040[Z14] - 1.3465[Z15] + 0.7267[Z16] + 0.7020[Z17] + 0.4269[Z18] + 0.3534[Z19] + 0.2938[Z20] - 0.6910[Z21] - 0.2516[Z22] - 0.3041[Z23] - 0.0474[Z24] + 0.3452[Z25] - 0.3854[Z26] + 0.4943[Z27] - 0.2350[Z28] - 0.1967[Z30] - 0.1633[Z31] - 0.9676[Z32] + 1.1317[Z33] + 0.5175[Z34] - 0.2339[Z35] - 0.5495[Z36] + 1.2304[Z37] + 0.1949[Z38] - 2.2098[Z39] + 1.4556[Z40] + 0.0003[Z41] - 2.5107[Z42] + 0.7512[Z43] - 1.0927[Z44] + 0.8470[Z45] + 2.0987[G1:A3] + 1.2444[G1:A3:A4] + 0.9489[G1:T2] + 0.8950[A3:A14] + 0.7286[T2:A3] + 0.6600[A4:A13:A14] + 0.5551[A3:A4:A15] + 0.4958[A4:A15] + 0.1673[A4:A14] - 0.3134[A3:A4:A14]$	0.000753333006677256
Cra	$\text{logit}[Pr(Y=1)] = -8.8761 - 0.2364[Z1] - 0.1567[Z2] + 0.2257[Z4] - 0.2514[Z8] + 0.0059[Z9] + 0.7806[Z11] + 0.0906[Z25] - 0.6701[Z26] - 0.3979[Z27] - 0.2360[Z28] - 0.0546[Z30] + 0.0183[Z33] - 0.1082[Z34] - 0.1364[Z35] - 0.4409[Z36] - 0.6281[Z38] - 0.1278[Z39] - 0.5138[Z42] + 0.3797[Z44] - 0.1857[Z45] + 0.0732[Z49] + 0.2873[Z51] - 0.8730[Z52] - 0.0203[Z53] - 0.0820[Z54] + 2.6341[T5:G6:A7] + 1.7954[G3:G6:A8] + 1.7789[A7:A8:T13] + 1.4899[A7:A8:C10] + 1.3445[A7:A8:A16] + 1.1704[T5:A8:C10] + 1.1321[A6:A7] + 0.7426[A7:A8:T14] + 0.6533[A7:A8:G11] + 0.4875[G6:C10] + 0.4513[C4:A7] + 0.4504[G3:A7:C10] + 0.3481[G3:G6:A7] + 0.0512[C4:T5] + 0.0366[G3:A8:T13] - 0.1465[A7:A8:T9] - 0.2314[G6:A7:A8] - 1.0269[G6:A8:T13]$	0.002713031341147840

TF	The ELRM	The optimal λ
CsgD	$\mathrm{logit}[Pr(Y=1)] = -9.4878 + 0.6654[Z1] + 0.3086[Z2] - 0.5520[Z3] + 0.5936[Z6] + 1.4485[Z7] - 0.5445[Z8] + 1.1742[Z9] + 1.1610[Z10] - 0.5643[Z11] + 1.3498[Z12] - 0.0462[Z14] + 1.2910[Z16] - 0.3222[Z17] + 0.2420[Z18] - 0.3724[Z20] - 0.5524[Z21] + 0.1997[Z22] - 0.1949[Z23] + 0.9377[Z24] + 0.4354[Z26] - 1.3494[Z27] - 0.2074[Z28] + 0.2063[Z29] - 0.3995[Z30] + 0.7659[Z31] + 2.2346[T10:A11] + 2.0273[G6:A11] + 1.7377[T5:A11] + 1.0818[A7:A11] + 0.2513[A7:A8] - 0.1143[A10:A11]$	0.002306349440630090
CysB	$\mathrm{logit}[Pr(Y=1)] = -10.2287 - 0.1269[Z2] - 0.5314[Z4] - 0.0337[Z5] + 0.8715[Z7] - 0.6886[Z11] - 0.4841[Z12] - 0.5476[Z14] - 0.1947[Z15] - 0.2260[Z19] - 0.7407[Z20] - 0.3668[Z22] - 0.6590[Z24] + 0.0595[Z26] + 0.2035[Z29] + 0.8943[Z31] + 0.2534[Z32] - 0.2476[Z35] - 0.3446[Z36] - 0.0085[Z38] - 0.2964[Z39] - 0.3593[Z40] - 0.5099[Z41] - 0.1897[Z44] + 0.0231[Z46] - 0.0446[Z49] - 0.3152[Z51] - 0.0225[Z54] - 0.0119[Z56] - 0.4902[Z57] - 0.0811[Z58] - 0.1627[Z59] - 0.5786[Z61] - 0.5413[Z62] - 0.7849[Z63] + 1.4095[Z65] + 0.0813[Z68] + 0.2298[Z69] - 0.5183[Z72] - 0.4624[Z73] - 0.3716[Z75] + 0.2968[Z76] + 0.4479[Z77] - 0.8013[Z79] - 0.2610[Z80] + 0.6248[Z83] + 0.5208[Z89] - 0.2326[Z92] - 0.2024[Z94] - 0.0666[Z96] - 0.0055[Z104] - 0.5005[Z105] + 0.0170[Z109] + 0.2393[Z111] + 0.6347[T5:A6]$	0.001729994744709460
CytR	$\mathrm{logit}[Pr(Y=1)] = -6.8158 - 0.1610[Z2] - 0.2338[Z3] - 0.1489[Z14] - 0.1012[Z22] - 0.2409[Z24] - 0.3030[Z38] - 0.1197[Z39] - 0.3601[Z40] - 0.0602[Z44] - 0.1119[Z53] + 1.7814[G9:C10:A11] + 1.6866[G9:T18] + 1.5752[T5:G9:A12] + 1.4408[G9:A12:T17] + 1.3113[T5:G9:A11] + 1.0920[G9:A11] + 1.0694[T8:A12] + 0.3711[G9:A11:T17] + 0.2556[T6:G9] + 0.1031[G9:A11:A12] + 0.0663[G9:C10:A12]$	0.009410092445941080
Dan	$\mathrm{logit}[Pr(Y=1)] = -6.3534 + 0.1783[Z1] + 2.6757[A5:T6:T7] + 2.1248[G1:A5:T6] + 1.9400[T2:T6:T7] + 0.7646[T2:A5:T6] + 0.4093[G1:T3:T6] + 0.4015[G1:T3:A5] + 0.1981[G1:T7] + 0.1235[T2:T3:A5] + 0.0866[T3:A5:T6] + 0.0210[G1:A5]$	0.013734948976763400
DcuR	$\mathrm{logit}[Pr(Y=1)] = -8.6948 - 0.6222[Z4] + 0.1151[Z9] - 0.0178[Z21] - 0.2111[Z31] - 0.2774[Z36] + 0.1214[Z46] + 1.7590[A8:T11] + 1.6371[T1:T5:A17] + 1.5646[T13:A17] + 1.5225[T1:A8:A17] + 1.3056[T1:T5:T10] + 1.1894[T7:A8] + 1.1611[T1:T5:A8] + 0.6203[T1:T10:A17] + 0.5584[T1:T5:T6] + 0.2822[T5:T6:A17] + 0.2693[T5:A9] + 0.1628[T5:A17]$	0.005575658955745730
DeoR	$\mathrm{logit}[Pr(Y=1)] = -6.0668 + 2.3235[T1:C7:A8] + 1.5618[A6:C7] + 1.0159[A6:A8] + 0.9638[C3:C7:A8] + 0.7592[T1:C3:C7] + 0.7551[T4:A8] + 0.6600[T1:C3:A8] + 0.1139[C7:A8:A11] + 0.0050[T1:C7]$	0.019203892496285400
DgsA	$\mathrm{logit}[Pr(Y=1)] = -9.0338 + 1.9933[T2:T6:G15] + 1.4771[T4:G15:A17] + 1.3000[T5:A17:A18] + 1.2277[T4:T6:G15] + 1.0830[T5:A18:T20] + 0.9555[T2:A3] + 0.8450[T2:T6:T7] + 0.8276[T2:T7:A17] + 0.7537[T1:T6:A18] + 0.6092[T1:T2:T6] + 0.5143[T5:G15:A18] + 0.3735[T2:G15:A18] + 0.2943[T6:G15:A18] + 0.2931[T4:A17:A18] + 0.2824[T4:T7:G15] + 0.2592[T5:A17:A21] + 0.1772[T4:G15:A18] + 0.0716[T5:A18:T23] + 0.0680[T2:T6:A18]$	0.004098024260995270
DnaA	$\mathrm{logit}[Pr(Y=1)] = -6.4395 + 1.9503[T4:C10:A11] + 1.1474[T6:A9:C10] + 0.9685[T6:C8:C10] + 0.9675[T4:T6:C8] + 0.9271[T4:T6:A9] + 0.8322[T3:A5:C10] + 0.8131[T4:A5:C10] + 0.6202[T4:C7:A11] + 0.5273[T4:C7:C10] + 0.3863[T4:A9:C10] + 0.3783[A1:T4] + 0.2858[T3:T4:C10] + 0.2524[T4:C8:A11] + 0.2200[T3:T4:A5] + 0.1803[T4:C8:C10]$	0.013284692566310900
EvgA	$\mathrm{logit}[Pr(Y=1)] = -10.8318 + 1.8847[G12:T13:A17] + 1.4161[C7:G16:A17] + 1.0878[G12:G16:A17] + 1.0555[T11:G12:T13] + 0.9127[T5:G12:T13] + 0.8321[A6:G12:G16] + 0.8144[C7:T13:A17] + 0.7713[T5:C7:G12] + 0.6639[C7:A14:G16] + 0.6362[C7:G12:T13] + 0.6124[A6:C7:T11] + 0.5685[G12:T13:A14] + 0.5559[G12:A14:G16] + 0.5522[A6:T11:A14] + 0.5269[T5:C7:A17] + 0.4625[T5:G12:A14] + 0.4293[T11:A14:G16] + 0.3764[C3:T5:G12] + 0.3634[T1:A6:C7] + 0.3453[C7:T11:T13] + 0.3395[A6:C7:A17] + 0.3091[A6:C7:G12] + 0.3052[C7:T11:A14] + 0.3001[A6:G12:A17] + 0.2293[A6:G12:A14] + 0.2257[T5:G16:A17] + 0.2197[T1:T13:A17] + 0.1704[A6:T11:G12] + 0.1166[A14:G16:A17] + 0.0812[C7:T13:A14] + 0.0141[T11:G16:A17] + 0.0085[T5:T11:A14]$	0.000397161009464120
ExuR	$\mathrm{logit}[Pr(Y=1)] = -10.4328 + 3.4499[T5:G7:T17] + 2.7078[T5:G7:A14] + 1.5219[A1:T5:G7] + 1.4240[A1:T5:T17] + 1.2841[G7:T8:A14] + 1.2105[T5:A14:T17] + 1.0354[A1:G7:T17] + 0.9685[T5:T8:A14] + 0.9204[G7:A14:T17] + 0.4821[A1:G7:A14] + 0.4573[T8:A14:T17] + 0.4231[A1:T8:T17] + 0.2035[A2:T5:A14] + 0.1786[A1:T8:A14] + 0.1658[A1:T5:A14] + 0.1035[A1:G7:A11] + 0.0805[A1:T5:T8] + 0.0172[A1:T5:A11] + 0.0144[A1:A14:T17]$	0.000402211874589739
FNR	$\mathrm{logit}[Pr(Y=1)] = -9.8605 - 0.0845[Z1] - 0.7743[Z2] - 0.1945[Z3] - 0.2046[Z4] - 0.1514[Z6] + 0.0910[Z7] + 0.6079[Z8] + 0.6568[Z9] + 0.5652[Z10] + 0.2300[Z11] + 0.0260[Z12] - 0.5879[Z14] - 0.5577[Z15] - 0.5085[Z16] + 0.2462[Z17] - 1.2642[Z18] + 0.2545[Z19] - 0.2110[Z20] + 0.8712[Z22] + 0.2013[Z26] - 0.1080[Z27] - 0.1860[Z29] - 0.0463[Z32] - 0.2921[Z33] - 0.3998[Z34] + 0.2336[Z36] - 0.0225[Z39] + 0.7970[Z41] - 0.5141[Z42] + 2.1271[C12:A13:A14] + 1.9008[A10:A13:A14] + 1.8389[A10:T11:C12] + 1.3442[T2:T11:A13] + 1.1120[T11:A13:A14] + 0.8988[G3:T11:A13] + 0.8414[T2:T11:C12] + 0.8019[T2:C12:A13] + 0.5521[T11:C12] + 0.5346[A10:A13] + 0.5204[T5:T11:A13] + 0.5180[T11:A13] + 0.3594[T5:A13] + 0.2852[T11:C12:A13] + 0.2851[C12:A14] + 0.2807[T11:C12:A14] + 0.2579[T2:A10:T11] + 0.2001[A8:T11:A13] + 0.1123[T2:A13] + 0.1064[A10:C12:A13] + 0.0327[T5:A13:A14]$	0.001288822297987360
FadR	$\mathrm{logit}[Pr(Y=1)] = -11.4663 - 0.1129[Z27] + 0.3616[Z38] - 0.0631[Z41] + 0.3384[Z44] + 4.1370[T4:G5:C8] + 2.6830[T7:A11] + 2.5534[C3:G5:G10] + 2.4213[G5:G6:G10] + 2.0989[A1:G5:G10] + 1.7945[G5:C8:T16] + 1.7894[G5:T7:T16] + 1.3443[G5:G6:T7] + 1.3073[G5:G10:T14] + 0.5936[G5:G10:A11] + 0.5788[C3:G10] + 0.5505[G5:A11] + 0.4802[A1:G5:T7] + 0.2700[G6:T7] + 0.2323[C3:T7]$	0.001421265353981790
FhlA	$\mathrm{logit}[Pr(Y=1)] = -7.5894 - 0.3235[Z1] - 0.1456[Z8] - 0.3152[Z18] + 0.2369[Z35] + 0.1997[Z37] + 0.2147[Z38] + 2.2145[C4:A8:G11] + 2.1693[C4:G5:A8] + 1.6277[C4:A6] + 1.4212[G2:C4:A8] + 1.3034[C1:C4:A8] + 1.1470[A6:A8] + 1.0197[A8:C9] + 0.6593[A6:A7] + 0.2833[T3:C4] + 0.2645[C4:G11] + 0.1913[A8:G11]$	0.007452771207372760
Fis	$\mathrm{logit}[Pr(Y=1)] = -11.4419 - 2.4964[Z1] + 0.2582[Z2] + 0.8105[Z3] + 0.0890[Z4] + 0.2107[Z5] + 0.0520[Z8] - 0.1071[Z9] - 0.4888[Z10] + 0.2225[Z11] - 0.2221[Z12] + 1.3194[Z13] - 0.8215[Z14] + 0.5695[Z15] + 0.8621[Z16] - 0.5340[Z17] - 1.0748[Z18] + 0.2550[Z19] - 0.9364[Z20] - 0.6421[Z21] - 2.1315[Z22] - 0.1572[Z23] - 0.0998[Z24] + 0.0901[Z25] - 0.4090[Z26] - 0.4187[Z27] + 0.6515[Z28] - 0.5178[Z29] - 0.9662[Z30] - 0.3648[Z31] + 0.6371[Z32] - 1.8810[Z33] + 0.5798[Z34] + 0.1278[Z35] + 0.7856[Z36] + 0.7477[Z37] - 0.0246[Z38] + 0.1245[Z39] + 0.1075[Z40] - 0.0753[Z41] + 0.0200[Z42] + 0.9822[Z44] - 1.2496[Z45] + 3.0745[G1:T8] + 2.9837[G1:C15] + 2.0166[T8:C15] + 0.6959[A5:C15] + 0.6376[T9:C15] - 0.1743[T8:T10] - 0.3669[T7:C15] - 0.5513[G1:T7:T8] - 2.6496[G1:T8:C15]$	0.000296190771500001
FlhDC	$\mathrm{logit}[Pr(Y=1)] = -8.9333 + 0.1317[Z2] + 1.0046[Z3] - 0.2881[Z4] - 0.9075[Z7] - 0.2989[Z8] - 0.5164[Z11] - 0.8830[Z12] - 0.3035[Z15] - 0.2595[Z16] - 0.1824[Z17] - 0.4836[Z18] - 0.1997[Z19] - 0.0938[Z21] + 0.2653[Z23] - 0.1698[Z24] + 0.0139[Z26] + 0.0875[Z27] + 0.3668[Z30] + 0.1346[Z32] + 0.0615[Z33] + 0.0534[Z35] + 0.4560[Z36] + 0.0754[Z38] + 0.7713[Z39] + 0.0985[Z40] - 0.3084[Z41] - 1.0198[Z42] - 0.4729[Z43] - 0.1903[Z45] - 0.1014[Z46] - 0.4129[Z47] - 0.7157[Z48] + 1.7938[G11:C12] + 1.4259[T7:G10] + 1.3616[C12:T15] + 1.0980[T7:T15] + 1.0330[T7:C12:T15] + 0.2287[T6:T7:T15]$	0.003673305218001970

TF	The ELRM	The optimal λ
Fur	$\text{logit}[Pr(Y=1)] = -8.9186 + 0.0177[Z1] + 0.1000[Z3] + 1.1627[Z4] - 0.2876[Z5] - 0.0771[Z9] - 0.2010[Z10] - 0.3169[Z11] + 0.9586[Z12] + 0.2187[Z13] + 0.2255[Z14] + 0.0544[Z15] - 0.7845[Z17] + 0.2673[Z18] - 0.0111[Z20] - 0.0849[Z21] + 0.3585[Z22] - 0.0847[Z24] - 0.2769[Z25] - 0.2380[Z28] - 0.0929[Z29] + 1.0887[Z30] + 0.8963[Z31] + 0.1769[Z33] - 0.1657[Z34] + 0.5841[Z37] - 0.3087[Z38] - 0.2215[Z39] + 0.1929[Z40] - 0.0279[Z42] - 0.2623[Z43] + 0.4319[Z47] - 0.6196[Z48] + 0.6077[Z49] + 0.0167[Z53] - 0.9700[Z54] + 1.2659[A1:A5:T13] + 1.0970[T3:A7] + 0.9472[A1:A11:T12] + 0.7203[T6:A7] + 0.6308[A1:A5:T12] + 0.6208[A1:T3:T13] + 0.5799[A1:A11:T13] + 0.5515[A1:T12:T13] + 0.5252[A11:T13] + 0.5120[A1:A7:T12] + 0.3955[A8:T12] + 0.3176[A1:T3:T12] + 0.2962[A1:T3] + 0.1501[A1:A5:A11]$	0.001938822286628000
GadE	$\text{logit}[Pr(Y=1)] = -13.7526 + 0.1077[Z25] - 0.1141[Z28] + 0.0503[Z37] + 0.2451[Z40] - 0.3101[Z54] + 4.4440[T1:A4:A13] + 2.7951[A4:T7:A12] + 1.8318[T1:T7:A12] + 1.6527[T1:A4:A12] + 1.6362[T7:A8:A12] + 1.5546[T7:A13] + 1.2597[A4:A6:A12] + 1.1552[A8:A13] + 0.9517[A4:A8:A12] + 0.4947[T1:A6:A12] + 0.4598[T7:A8] + 0.4163[T1:A13] + 0.4007[A4:T7]$	0.000362966805881512
GadW	$\text{logit}[Pr(Y=1)] = -11.5367 + 0.9781[Z1] + 1.0101[Z2] - 0.0569[Z3] - 0.1160[Z4] - 0.3541[Z5] + 0.0361[Z7] - 0.0195[Z8] + 0.6764[Z9] + 0.9294[Z10] - 0.3161[Z11] - 0.6298[Z12] - 0.8078[Z13] - 0.6437[Z15] - 0.5522[Z17] + 0.3783[Z18] - 1.2160[Z19] + 0.3362[Z22] - 1.1274[Z23] - 0.3134[Z24] - 0.7338[Z26] + 0.0202[Z27] + 0.2845[Z28] - 0.6515[Z29] - 0.8774[Z30] - 1.1392[Z31] + 1.0070[Z32] - 1.3708[Z33] + 0.0113[Z34] + 0.2699[Z35] + 0.4252[Z36] - 0.2407[Z38] - 0.6180[Z39] - 0.2042[Z40] + 0.0009[Z41] - 0.5258[Z42] + 0.4038[Z43] - 0.6962[Z44] - 0.4771[Z45] - 0.3027[Z46] - 0.7508[Z47] + 0.3670[Z49] - 0.6563[Z51] + 1.4073[Z52] + 1.0273[Z53] - 0.2014[Z54] - 0.9477[Z56] - 1.1087[Z59] + 1.1602[Z60] + 1.9642[T5:A9]$	0.001594161167225190
GadX	$\text{logit}[Pr(Y=1)] = -9.3199 - 1.0692[Z2] + 0.6711[Z4] - 0.9605[Z6] + 0.2948[Z7] + 0.0725[Z8] - 0.5884[Z11] + 0.0661[Z12] - 0.2139[Z14] + 0.9505[Z15] - 1.0791[Z16] + 0.0173[Z17] - 0.3012[Z18] + 0.1415[Z19] + 0.6148[Z20] + 0.1655[Z21] - 0.2366[Z22] - 0.5161[Z23] - 0.0347[Z25] - 1.6634[Z26] + 0.4637[Z27] + 0.9309[Z28] + 0.2466[Z29] - 0.6297[Z32] + 0.2013[Z34] - 0.4986[Z35] + 0.0157[Z36] - 0.7090[Z37] - 0.2134[Z38] - 0.4629[Z40] - 0.2317[Z42] - 0.0515[Z44] + 0.4165[Z46] + 0.1597[Z47] + 0.0059[Z50] - 0.2578[Z51] + 0.4618[Z52] - 0.1657[Z53] - 0.3727[Z56] - 0.2950[Z57] + 1.7830[A3:G5] + 1.6361[A3:T11] + 0.8645[A2:T13] + 0.8402[A10:T13] + 0.4484[A1:A2] + 0.1484[A3:T13]$	0.002709319450276420
GalR	$\text{logit}[Pr(Y=1)] = -6.7042 + 0.5367[Z13] - 0.1271[Z16] - 0.2884[Z18] + 0.2164[Z19] - 0.3570[Z29] + 0.2527[Z34] + 0.0643[Z39] - 0.0868[Z43] - 0.1658[Z45] + 2.2163[G1:A4:T9] + 1.3624[A3:A4:A13] + 1.3571[A13:C14] + 0.7840[A4:T9:C12] + 0.7451[G1:A3:A4] + 0.6523[A3:A4:G7] + 0.6360[G1:A4:C6] + 0.6132[G1:A4:C12] + 0.5692[A4:C6:T9] + 0.5373[A4:G7:A13] + 0.4803[A2:A3] + 0.2789[G1:A4:A13] + 0.1120[A3:A4:T9]$	0.008256818119450710
GalS	$\text{logit}[Pr(Y=1)] = -9.3140 + 0.6713[Z1] + 0.1257[Z3] - 0.0307[Z12] - 0.0960[Z22] - 0.0069[Z34] - 0.1300[Z35] + 0.0998[Z39] + 0.3586[Z40] - 0.0544[Z44] + 0.1915[Z45] + 2.2620[A6:G9:C14] + 1.4103[T2:G3:A6] + 1.3674[G3:A5:A15] + 1.2944[T2:A6:T11] + 1.2181[G3:A5:T11] + 1.0900[G3:A6:T11] + 0.9159[G3:A5:A6] + 0.8728[A4:A6:T11] + 0.8288[G3:T11:C14] + 0.6347[A5:A6:T11] + 0.5828[T2:G3:A5] + 0.5798[T4:A5] + 0.5720[A5:A6:A15] + 0.4958[G3:T11:A15] + 0.4119[G3:A6:A15] + 0.4011[G3:A5:C14] + 0.3257[T2:A5:A6] + 0.3078[A5:T11:A15] + 0.2803[G3:A6:C8] + 0.2666[T2:A5:T11] + 0.0279[A6:C8:T11] + 0.0174[A5:A6:C8] + 0.0160[A6:T11:A15] - 0.5343[C1:G3]$	0.002563811143001620
GcvA	$\text{logit}[Pr(Y=1)] = -11.6813 - 0.5027[Z7] - 0.3493[Z8] + 0.0424[Z13] - 0.0366[Z16] - 0.0176[Z17] + 0.4662[Z20] + 0.2290[Z28] - 0.1073[Z31] + 0.1841[Z36] + 0.0449[Z42] - 0.0152[Z47] + 0.4820[Z49] + 0.5912[Z59] + 3.2230[T11:A18] + 2.8293[A15:A18:T24] + 2.6364[A15:A18:A22] + 2.2229[A22:T24] + 2.0689[T11:A15] + 1.2036[A16:A17] + 0.2273[T21:A22]$	0.000854422737643911
GlpR	$\text{logit}[Pr(Y=1)] = -18.2562 + 0.4804[Z1] + 0.3296[Z4] - 0.9261[Z5] - 0.6253[Z6] + 1.8402[Z7] - 0.4790[Z9] - 1.6312[Z10] - 0.5806[Z11] - 1.2069[Z12] - 0.2380[Z13] - 1.1863[Z14] + 1.1093[Z15] + 0.4022[Z16] - 0.0689[Z17] - 1.0670[Z18] - 0.6262[Z19] + 0.0351[Z20] + 0.2050[Z21] + 0.5788[Z23] + 1.4229[Z24] + 1.3437[Z25] + 0.3087[Z27] + 0.8229[Z28] - 0.3093[Z29] + 0.3367[Z31] - 1.9724[Z32] - 0.1465[Z33] - 0.5008[Z36] + 0.6728[Z37] - 0.1104[Z38] - 1.1594[Z39] - 1.1495[Z40] + 2.5356[Z41] - 0.3498[Z42] + 0.1810[Z43] - 0.5929[Z44] + 1.2237[Z45] + 0.0899[Z46] + 1.6446[Z47] + 1.9145[Z49] + 2.2855[Z53] + 0.8780[Z55] - 1.1224[Z56] + 0.0661[Z57] - 0.9258[Z60] + 4.5039[G15:A16] + 4.2298[G5:C8] + 2.6646[G17:C18] + 1.5530[G15:C16]$	0.000151862470372034
GntR	$\text{logit}[Pr(Y=1)] = -6.0260 + 0.0517[Z29] + 1.9570[T5:T12:C15] + 1.5943[T3:T5:T12] + 1.2873[T5:T12:A13] + 1.1557[T5:T12:A16] + 0.6434[G4:T5] + 0.6292[T5:A13:C15] + 0.6032[T5:A7:T12] + 0.3479[T6:A7] + 0.3281[C10:T12]$	0.018712648472423100
HNS	$\text{logit}[Pr(Y=1)] = -13.5813 - 0.6682[Z1] - 0.9663[Z2] + 0.9961[Z3] + 2.0186[Z4] - 1.0180[Z5] + 0.6558[Z6] + 2.6597[Z7] + 0.9017[Z8] - 0.2767[Z9] + 1.1203[Z10] - 0.6547[Z11] - 2.4222[Z12] + 2.0446[Z13] - 1.3158[Z14] - 2.1585[Z15] + 1.9624[Z16] - 2.9765[Z17] - 0.2956[Z18] + 0.9248[Z19] - 2.7649[Z20] + 1.1617[Z21] + 0.4210[Z22] + 0.0017[Z23] - 0.1286[Z24] - 3.0454[Z25] + 2.0537[Z26] - 0.6757[Z27] - 1.2546[Z28] + 1.1772[Z29] + 1.5073[Z30] + 1.2572[Z31] + 0.3726[Z32] - 1.9088[Z33] + 0.9393[Z34] + 0.1339[Z35] + 1.2880[Z36] - 0.2280[Z38] - 0.1247[Z39] + 2.3217[A3:A13] - 0.3828[A3:A11]$	0.000433154195809349
HipB	$\text{logit}[Pr(Y=1)] = -10.3749 + 2.4115[C5:G15:A16] + 1.8609[T3:T17:A19] + 1.6390[T17:A18:A19] + 1.5489[T3:G15:A18] + 1.4517[T3:A16:A19] + 0.8037[C4:T17:A19] + 0.7033[G15:T17:A18] + 0.6867[T3:G12:G15] + 0.6691[T3:G12:A19] + 0.5579[A2:G15:A19] + 0.5332[G12:G14:A18] + 0.5272[A2:G12:A19] + 0.4537[T1:G15:A19] + 0.4383[T3:G15:A19] + 0.3755[G14:A16:A19] + 0.3354[C4:G12:A19] + 0.3299[G12:A18:A19] + 0.3259[G12:G14:A19] + 0.2823[C4:G12:A16] + 0.1524[C4:A16:A19] + 0.1303[G12:A16:A18] + 0.1122[T1:G15:T17] + 0.0954[A2:T3:A19] + 0.0367[T1:G12:A18] + 0.0278[G15:A16:A18]$	0.000467384119465767
IHF	$\text{logit}[Pr(Y=1)] = -11.5843 + 0.1199[Z1] - 0.7866[Z2] + 0.0305[Z3] + 0.5510[Z4] - 0.5883[Z5] - 0.0435[Z6] + 0.3484[Z7] + 1.2089[Z8] - 1.4827[Z9] - 1.0109[Z10] + 0.2875[Z11] + 0.0397[Z12] - 1.0174[Z13] + 0.5692[Z14] + 0.0543[Z15] + 0.0607[Z16] + 0.2757[Z17] + 0.4918[Z18] + 0.3216[Z19] - 0.1483[Z20] - 0.1465[Z21] - 0.0934[Z22] + 0.3340[Z23] - 0.8275[Z24] + 0.4752[Z25] - 0.9265[Z26] - 1.0399[Z27] - 1.2958[Z28] - 1.9865[Z29] + 2.2871[Z30] + 1.3139[Z31] - 0.3396[Z32] - 1.5034[Z33] + 1.4101[Z34] - 1.0489[Z35] - 2.4359[Z36] + 0.5856[Z37] - 0.6713[Z38] - 0.8801[Z39] + 1.7015[T1:T9] + 1.6105[A2:A3:T9] + 1.5451[A3:T9:G10] + 1.3096[A3:T12] + 0.9074[A3:T9] + 0.5606[A2:T9] + 0.4491[A2:T9:T12] - 1.4530[A3:T9:T12]$	0.000471837595689312
IclR	$\text{logit}[Pr(Y=1)] = -5.2691 + 3.4105[T1:T3:A6] + 1.5336[T1:T2:T7] + 0.4336[T1:T2:T3] + 0.3330[T1:C5] + 0.3321[T1:T3:T4] + 0.2560[T2:A6] + 0.1299[T3:A6] + 0.1058[T1:T2]$	0.021320936257994600

TF	The ELRM	The optimal λ
IscR	$\mathrm{logit}[Pr(Y=1)] = -21.3486 - 0.4022[Z2] - 1.3011[Z3] + 1.8973[Z4] - 0.5507[Z6] + 0.8620[Z7] - 1.2722[Z8] - 0.7438[Z9] + 0.8118[Z10] + 1.2279[Z11] - 0.9713[Z12] + 1.2391[Z14] - 0.0043[Z15] + 3.0620[Z17] + 0.6249[Z20] - 0.4194[Z21] + 0.4136[Z22] - 1.3998[Z23] - 0.4165[Z24] + 0.3301[Z25] - 0.9570[Z26] + 1.5883[Z29] + 0.7837[Z30] - 1.2594[Z32] - 1.1368[Z33] + 1.0027[Z34] - 0.9222[Z35] - 0.1994[Z36] + 2.5647[Z37] - 0.0395[Z39] - 0.7368[Z40] - 0.6693[Z44] - 0.3934[Z45] - 1.8962[Z46] - 0.2653[Z47] + 1.3685[Z49] - 0.1499[Z50] + 0.5963[Z51] + 0.0032[Z52] - 0.2132[Z53] + 1.5260[Z54] - 1.1879[Z55] - 2.0710[Z56] - 0.0511[Z57] + 1.3327[Z60] + 1.0562[Z61] + 1.2566[Z63] + 0.6536[Z64] - 0.2830[Z65] - 1.1186[Z66] + 5.2668[C7:G9] + 4.8453[A2:C5] + 3.6442[A11:A12] + 1.1307[T1:A2]$	0.000023457300416545
LeuO	$\mathrm{logit}[Pr(Y=1)] = -11.8161 + 3.8611[T3:T13:T15] + 3.7533[T2:T3:T13] + 1.6051[T3:G8:T18] + 1.5435[T2:T3:G8] + 1.3180[T13:T15:T18] + 1.2271[T3:T15:A17] + 0.9281[T2:T15:A17] + 0.9067[G8:T15:T18] + 0.6834[T2:T15:T18] + 0.6153[T2:T3:A17] + 0.5726[T2:A17:T18] + 0.5402[T2:T3:T15] + 0.2278[T13:A17] + 0.0870[T2:T3:T18] + 0.0492[T3:A17:T18] + 0.0135[T2:G8]$	0.000292842894204757
LexA	$\mathrm{logit}[Pr(Y=1)] = -7.3733 - 0.0279[Z18] + 1.5673[C3:T8:A11] + 1.2138[T4:G5:C16:G18] + 1.0769[A2:C3:C16] + 1.0663[C3:T4:A12] + 0.8846[C3:T4:G5:A17] + 0.8044[T4:G5:A11:C16] + 0.7914[C3:G5:C16:A17] + 0.7640[C3:G5:A17:G18] + 0.6370[T4:A7:C16] + 0.5669[C3:T4:C16:G18] + 0.5538[C3:C16:A17:G18] + 0.4614[G5:A11:C16:G18] + 0.4105[C3:G5:T8] + 0.3984[A2:C3:T4] + 0.3910[A2:C3:G5] + 0.3311[C3:T4:T8] + 0.3118[C3:A9:A11] + 0.3025[T4:A17:G18] + 0.2722[C3:A11] + 0.2460[C3:T4:A11] + 0.2022[C3:A11:A15] + 0.1807[C3:T4:G5:A7] + 0.1291[C3:A11:C16:G18] + 0.1184[G5:A7:C16] + 0.0859[T4:C16:A17] + 0.0459[G5:A7:A11] + 0.0424[C3:T4:G5:A9] + 0.0165[C3:G5:A11:A17] + 0.0098[G5:A9:C16] + 0.0044[C3:G5:A11]$	0.005549007334489950
Lrp	$\mathrm{logit}[Pr(Y=1)] = -8.5684 + 0.0238[Z1] + 0.6906[Z2] + 0.0436[Z3] + 0.9507[Z4] - 1.2175[Z5] - 0.0071[Z6] - 0.7362[Z7] - 1.0245[Z8] + 0.2416[Z9] - 2.0329[Z11] + 0.6665[Z12] - 0.0267[Z14] - 0.5238[Z15] - 1.1729[Z16] + 0.4173[Z17] - 0.2775[Z18] + 1.4346[Z19] - 0.5015[Z20] - 0.9843[Z21] - 0.5209[Z22] - 0.4624[Z23] - 0.3452[Z24] - 0.3226[Z25] - 0.2502[Z26] + 0.8988[Z27] - 0.2153[Z28] + 1.5046[Z29] - 1.0435[Z30] - 0.4415[Z31] - 0.2288[Z32] + 0.0236[Z33] - 1.0770[Z34] + 0.4641[Z36] + 1.1086[C10:G12] + 1.0240[T5:T8] + 0.8275[T5:T11] + 0.7216[C10:T11] + 0.3241[T6:T8] + 0.3064[T8:C10] - 0.0329[T4:T8] - 0.4655[T5:C10] - 0.6618[T3:T8]$	0.000918279013539920
MalT	$\mathrm{logit}[Pr(Y=1)] = -8.5759 + 0.1935[Z1] + 0.2505[Z2] + 0.1210[Z6] - 0.0974[Z29] + 2.3276[G5:G7] + 1.9514[T4:G5:G8] + 1.6387[G5:G8:A9] + 1.5482[G5:A6:G8] + 1.5398[T7:G10] + 1.4933[G4:G8:A9] + 1.4287[G4:G5:A9] + 1.3544[G3:G5:G8] + 1.3324[G5:G8:G10] + 1.1321[T7:G8:A9] + 0.3700[G4:G5:G8] + 0.3195[G4:G7] + 0.2243[A9:T10] + 0.1514[G7:G10] + 0.0485[G8:C9]$	0.005000458890918480
MarA	$\mathrm{logit}[Pr(Y=1)] = -9.5370 + 0.3489[Z1] - 0.7219[Z2] - 0.2299[Z4] - 0.1690[Z5] + 0.1609[Z6] - 0.1474[Z7] - 0.1578[Z9] - 0.4116[Z10] - 0.0593[Z11] + 0.4009[Z12] + 0.3999[Z13] + 0.4213[Z14] + 0.0296[Z16] + 0.1719[Z17] + 0.1841[Z18] + 0.2334[Z20] + 0.1722[Z22] + 0.2993[Z23] + 0.3838[Z25] + 0.2506[Z27] - 0.8328[Z29] - 0.5531[Z30] + 0.0640[Z31] - 0.3987[Z32] - 0.2905[Z33] - 1.1391[Z36] - 0.5009[Z38] + 0.9533[Z39] - 0.2139[Z41] - 0.7356[Z42] + 0.1204[Z44] + 0.1094[Z45] + 0.0271[Z47] - 0.8868[Z49] + 0.3814[Z50] - 0.5387[Z51] + 0.6202[Z52] - 0.1620[Z53] - 0.0228[Z55] - 0.1680[Z57] + 2.9488[G15:C16:T19] + 2.0353[T3:G15] + 1.8298[G18:T19] + 1.6251[T4:G15] + 0.8318[T14:G15:T19] + 0.5283[G15:T19] + 0.3646[T4:G15:T19] + 0.1917[T10:G15:T19]$	0.002381291606013950
MelR	$\mathrm{logit}[Pr(Y=1)] = -6.2107 + 2.2242[C6:C15:G17] + 1.5750[C15:G17:A18] + 1.0360[A11:C15:A18] + 0.7793[T3:C15:G17] + 0.7694[C6:A11:C15] + 0.6975[A11:A12:G17] + 0.5916[A11:A12:C15] + 0.5865[T3:C6:C15] + 0.5607[C6:A11:A18] + 0.2350[A11:C15:G17]$	0.018202411466757900
MetJ	$\mathrm{logit}[Pr(Y=1)] = -6.3095 + 0.0847[Z1] + 0.8821[Z3] - 0.1222[Z17] + 0.2549[Z20] + 2.7538[G2:A3:G5] + 1.5929[G2:A3:T4] + 1.2406[A3:G5] + 1.1888[A3:T6] + 0.9689[G5:T6] + 0.7625[A3:A4] + 0.6751[A3:A5] + 0.2394[A1:G2] + 0.2036[G2:A3]$	0.006976764127759090
MetR	$\mathrm{logit}[Pr(Y=1)] = -6.3661 - 0.0037[Z40] + 2.4844[A3:A4:C12] + 1.5398[A4:A7:C12] + 1.2811[A3:A7:C12] + 1.0595[C12:T14] + 0.7146[A3:A4:A7] + 0.7007[G2:A3] + 0.5411[A7:T9] + 0.0958[A3:A4:T10]$	0.016969176371332000
MlrA	$\mathrm{logit}[Pr(Y=1)] = -12.8112 + 0.5967[Z21] - 0.2676[Z24] + 0.0434[Z31] - 0.0416[Z32] + 0.2159[Z46] + 0.6490[Z53] - 0.3934[Z58] - 0.0240[Z59] + 0.5101[Z64] + 0.3979[Z75] - 0.5838[Z77] + 0.0134[Z83] + 0.5877[Z85] + 7.3404[A2:A27:A30] + 6.9406[A3:A4:G25] + 2.1170[G7:T8]$	0.000128256947504967
MntR	$\mathrm{logit}[Pr(Y=1)] = -10.7830 + 2.5469[A6:G7:C8] + 1.6257[A6:C8:G13] + 1.4976[G7:A16:T19] + 1.2642[T5:G7:A16] + 1.2596[A6:G13:T19] + 1.1070[T5:G7:C14] + 1.1039[C8:T15:T17] + 1.0766[C14:A16:T19] + 0.9802[T3:C8:A16] + 0.8510[C8:T15:T19] + 0.8205[A6:G13:T17] + 0.4907[C8:A16:T19] + 0.4671[G7:G13:T19] + 0.3459[T5:G7:G13] + 0.2798[G7:T17:T19] + 0.2354[T5:C14:A16] + 0.2091[C14:T15] + 0.1951[T5:G13:T19] + 0.1932[G13:A16:T19] + 0.1910[G7:C14:T19] + 0.1658[G7:C14:A16] + 0.1448[G7:G13:T17] + 0.1206[A6:G7] + 0.0550[T5:G7:T17]$	0.000375182350923026
ModE	$\mathrm{logit}[Pr(Y=1)] = -7.4081 - 0.2461[Z24] - 0.0872[Z47] + 2.3214[T4:A17:G24] + 1.5429[G5:T6] + 1.4710[T4:T6:G24] + 1.3735[T4:T6:A17] + 1.1993[T4:T6:A9] + 1.1702[T4:A9:G24] + 1.1296[G2:T6:G24] + 0.7052[G2:T4:T6] + 0.2990[T4:T6:C23] + 0.2952[G2:A17:G24] + 0.2836[G2:T6:A9] + 0.1323[T6:A17:G24] + 0.0832[T6:C23:G24] + 0.0163[G2:T6:A17]$	0.007984473833374610
MqsA	$\mathrm{logit}[Pr(Y=1)] = -10.2444 + 0.2884[Z1] + 0.0521[Z2] - 0.1095[Z9] - 0.0106[Z18] + 0.0472[Z25] + 0.0393[Z27] - 0.1246[Z28] - 0.1603[Z29] - 0.0754[Z30] + 0.7317[Z34] + 0.5513[Z36] - 0.1496[Z41] + 3.0913[A7:T10] + 2.5454[A7:G8] + 2.5326[A7:G11] + 2.0269[G9:T10] + 1.9685[T6:G8] + 1.5324[T3:A7] + 0.9864[T6:A7] + 0.7688[G11:T14] + 0.6828[C2:G4]$	0.001984572715018160
MqsAMqsR	$\mathrm{logit}[Pr(Y=1)] = -8.2968 + 2.5639[A1:A2:T16] + 2.0064[A1:A2:T9] + 1.5005[A2:T9:T16] + 1.4996[A2:T11:T16] + 1.4675[A1:A2:T11] + 1.2991[A1:T11:T16] + 0.5702[T9:T11:T16] + 0.4633[A2:T9:T11] + 0.3859[A1:T9:T11] + 0.2081[T9:A13] + 0.1323[A1:A2:G15]$	0.003610304072891120
Nac	$\mathrm{logit}[Pr(Y=1)] = -11.0195 + 0.3446[Z1] - 0.3457[Z2] - 0.3578[Z3] + 0.9336[Z4] - 2.1300[Z5] - 0.6466[Z6] + 0.6640[Z7] - 1.2072[Z8] - 0.1052[Z9] - 0.7443[Z10] + 0.7784[Z11] - 0.6169[Z12] - 0.4502[Z13] - 0.9710[Z14] + 0.7796[Z15] + 0.9772[Z16] + 0.7414[Z17] - 0.8885[Z19] - 0.9149[Z20] + 1.3901[Z21] + 0.2153[Z22] - 0.0898[Z24] + 0.6976[Z25] - 0.2967[Z26] - 0.6390[Z27] - 0.4710[Z28] - 0.6654[Z29] - 0.4233[Z30] + 0.9884[Z31] - 0.9142[Z32] + 0.6143[Z33] - 1.0192[Z34] + 0.0694[Z36] - 0.5028[Z37] + 0.7286[Z38] + 0.3398[Z40] + 0.8691[Z41] + 0.4571[Z42] - 0.9540[Z43] + 0.2410[Z44] - 0.7052[Z45] + 2.8226[T10:G13] + 2.4801[T4:T7:T10] + 1.1977[T10:A14] + 1.1727[T7:T10]$	0.001090391384830090
NagC	$\mathrm{logit}[Pr(Y=1)] = -8.7321 + 0.3962[Z1] + 0.1487[Z5] - 0.1126[Z8] - 0.0060[Z11] - 0.0575[Z12] + 0.0045[Z32] + 0.2109[Z34] + 0.1901[Z36] + 0.0106[Z41] + 0.0214[Z42] - 0.2225[Z46] + 0.6849[Z51] + 0.1134[Z60] - 0.7057[Z62] + 0.3634[Z66] + 2.6942[T4:T8:A19] + 2.3094[T8:G10] + 2.2427[T7:T8:A18:A19] + 1.5164[A5:T8:A19] + 1.2875[T7:G17:A19] + 1.1684[T21:A22] + 0.7406[T7:A19:A22] + 0.4448[T8:A18:A22] + 0.3707[T6:T8:A18] + 0.0142[T4:A5]$	0.004149338983312960
NanR	$\mathrm{logit}[Pr(Y=1)] = -11.0578 + 5.1371[A1:G4:G5] + 4.2538[A3:G4:G5] + 3.8317[A1:A3:G4] + 3.8166[A1:A3:G5] + 0.1547[C2:A3:G5] + 0.1514[C2:A3:G4]$	0.000281627916205183

TF	The ELRM	The optimal λ
NarL	$\mathrm{logit}[Pr(Y=1)] = -12.1356 + 0.6711[Z1] - 1.4066[Z2] - 0.0152[Z3] + 0.8240[Z4] - 0.2691[Z5] + 0.0566[Z6] - 0.3207[Z7] - 3.8062[Z8] + 2.0945[Z9] + 0.3567[Z10] - 0.4516[Z11] + 0.9811[Z12] + 1.9084[Z13] + 2.3918[Z15] - 2.7126[Z17] + 1.7458[Z18] - 1.0239[Z19] + 0.6604[Z21] + 1.4407[Z22] - 0.4352[Z23] - 0.5986[Z24] + 4.9991[T7:A8] + 3.5209[T7:T8] + 1.6104[C7:A8] + 0.7097[G6:T7] + 0.4160[A2:T7] - 0.6870[T3:T7]$	0.000272691975208015
NarP	$\mathrm{logit}[Pr(Y=1)] = -14.3966 + 0.7582[Z1] + 0.1594[Z2] - 0.9919[Z3] + 0.5867[Z7] + 0.3018[Z10] + 0.1271[Z11] - 1.3592[Z13] - 0.9912[Z15] + 0.4360[Z16] + 0.2942[Z17] - 1.0264[Z18] - 0.8030[Z19] + 1.7056[Z20] - 1.3626[Z21] - 0.7679[Z22] - 0.1364[Z23] + 4.4571[T2:A3] + 3.6846[T2:G3:C4] + 3.6546[T2:C5] + 3.5665[C1:T2] + 2.3643[A3:C4] + 1.9975[A1:C4] + 1.9830[T2:C4] + 1.9027[T2:T8] + 1.4981[T2:A4] + 1.3111[T2:C4:C5] + 0.5615[G3:C4]$	0.000796733193102565
NhaR	$\mathrm{logit}[Pr(Y=1)] = -7.3493 + 0.0002[Z1] - 0.1510[Z8] + 0.0818[Z15] - 0.7279[Z23] - 0.1208[Z24] + 0.0645[Z34] + 0.0041[Z45] + 2.8284[C4:T9:T11] + 2.4028[G6:T9:T11] + 2.0677[T9:T11:C13] + 1.5928[C4:T9:T10] + 1.4189[T11:A12] + 1.1341[C13:T14] + 0.3705[T3:G6] + 0.2901[G6:T9] - 0.4585[T11:T12]$	0.008040464651531750
NrdR	$\mathrm{logit}[Pr(Y=1)] = -11.0811 + 2.8447[C3:T8:G12] + 2.7602[C3:T8:T10] + 2.5094[A2:C3:G12] + 2.1376[A2:T8:G12] + 1.3084[C3:A7:T10] + 1.2790[A2:C3:T8] + 0.9665[T8:T10:G12] + 0.9566[A7:T8:T10] + 0.8060[A2:A5:G12] + 0.5139[A2:C3:A7] + 0.5092[A5:T8:G12] + 0.5075[A2:T8:T10] + 0.2486[A2:A7:T8] + 0.2084[A2:A7:T10] + 0.0971[A2:A7:G12]$	0.000321394714873135
NsrR	$\mathrm{logit}[Pr(Y=1)] = -18.4857 - 1.3962[Z2] - 2.0140[Z3] + 0.2641[Z7] - 0.5150[Z11] - 0.6267[Z12] + 0.2192[Z16] + 0.5412[Z20] - 0.6800[Z23] - 0.8002[Z26] - 1.4200[Z27] + 0.4198[Z28] - 1.7684[Z30] + 0.3281[Z31] - 0.1708[Z32] - 0.9255[Z33] + 4.1269[A2:A4:G6] + 3.7077[T5:C7] + 3.2073[A4:T5:G6] + 2.7578[A2:G6:A8] + 2.1742[A2:A4:T5] + 2.1004[A1:A2:T11] + 2.0363[A2:T5:G6] + 1.7999[A2:T5:A8] + 1.6206[A4:G6:T11] + 1.4768[A1:A2:T5:G6] + 1.3563[A1:A4:G6] + 1.3019[A2:G6:T10] + 1.2821[A1:A2:G6] + 0.9585[A1:A4:T11] + 0.8928[A2:A4:T11] + 0.8588[A2:T5:T10] + 0.8424[A1:A2:A4:T5] + 0.7200[A1:G6:T11] + 0.6975[A1:A2:A4] + 0.6855[A2:G3] + 0.5772[A1:T5:T10] + 0.4711[A4:G6] + 0.3520[A2:A4:T10] + 0.3212[A4:T5:T11] + 0.1851[A1:A2:T5] + 0.1397[A1:G6] + 0.1313[A2:A4] + 0.0600[A1:T5:T11]$	0.000176986414948770
NtrC	$\mathrm{logit}[Pr(Y=1)] = -10.5561 - 0.4068[Z2] - 0.2731[Z3] + 0.5222[Z8] + 0.2097[Z10] - 0.0271[Z13] + 0.0245[Z14] + 0.4301[Z17] - 0.3592[Z18] + 0.1939[Z19] - 0.7701[Z20] + 0.8938[Z23] + 0.3685[Z25] - 0.3069[Z26] + 0.2728[Z27] - 0.2424[Z31] - 0.5239[Z32] - 0.5590[Z33] + 0.6449[Z35] + 1.1533[Z36] - 0.2817[Z37] - 0.5654[Z38] - 0.3656[Z40] - 0.0227[Z41] + 0.1786[Z43] - 0.2139[Z48] - 0.3550[Z50] + 4.2775[G2:C5] + 2.9620[A7:G15:C16] + 1.9760[G13:C16] + 1.4486[A10:G13:G15] + 1.2862[A10:G15:C16] + 0.9757[C3:A10:G13] + 0.9538[C3:A10:C16] + 0.8908[A10:T11:G15] + 0.6494[C3:G13:G15] + 0.4955[C3:A10:G15]$	0.001418108702516330
OmpR	$\mathrm{logit}[Pr(Y=1)] = -12.4703 - 0.6800[Z2] - 0.5650[Z3] + 1.0103[Z4] - 0.9627[Z9] + 0.4408[Z10] + 0.6193[Z11] + 0.2976[Z12] - 1.2325[Z13] - 0.2876[Z15] - 1.5906[Z16] - 0.9609[Z17] + 2.2058[Z18] - 0.1549[Z20] - 1.5013[Z21] + 1.4868[Z22] - 1.0811[Z23] - 0.2072[Z24] + 0.6763[Z25] - 0.7048[Z27] + 0.9040[Z29] - 0.4883[Z30] + 0.3275[Z32] - 0.2318[Z33] + 1.7445[Z34] - 0.8746[Z35] - 0.5187[Z36] + 0.2258[Z37] + 0.8124[Z38] - 0.1080[Z39] + 0.8942[Z40] - 0.5370[Z41] + 0.7723[Z42] + 0.2965[Z43] - 0.4328[Z44] - 0.5489[Z45] - 0.5679[Z48] + 0.0268[Z49] - 0.6016[Z50] - 1.8342[Z51] + 0.3372[Z52] - 0.1469[Z53] + 0.7971[Z55] - 0.7501[Z56] - 0.8720[Z57] + 2.3420[A9:C10] + 0.5530[T5:A9]$	0.001008403992884600
OxyR	$\mathrm{logit}[Pr(Y=1)] = -12.2875 - 0.0105[Z3] + 0.6790[Z4] + 0.1240[Z6] + 0.6281[Z7] + 0.8344[Z9] - 0.1533[Z10] - 0.0147[Z12] - 1.4963[Z15] + 0.5249[Z16] - 0.2289[Z17] - 0.2777[Z18] - 0.0208[Z19] - 0.0396[Z21] + 0.1815[Z22] + 0.4490[Z24] + 0.2148[Z25] + 0.8040[Z26] - 0.4475[Z27] + 0.1923[Z29] - 1.3412[Z30] - 0.0859[Z31] - 1.3242[Z32] - 0.2297[Z33] + 0.4998[Z34] + 0.3171[Z35] + 0.4685[Z36] - 0.2367[Z37] - 0.2306[Z38] - 0.1398[Z39] - 1.0277[Z40] - 0.6326[Z42] + 1.1080[Z43] - 1.5383[Z44] + 0.3042[Z45] - 0.0898[Z46] + 0.6026[Z47] - 0.5387[Z48] + 0.3700[Z49] - 1.3016[Z50] - 0.2336[Z51] + 2.5031[G2:T13] + 1.9988[A8:T13] + 1.8733[A12:T13] + 1.7208[C10:A12] + 1.2953[T13:C14] + 0.8607[T4:T13] + 0.6377[A12:T13:C14] + 0.2216[T4:A12:T13] + 0.0656[T11:A12]$	0.001392670158356010
PdhR	$\mathrm{logit}[Pr(Y=1)] = -7.1660 + 0.0070[Z1] + 0.2232[Z4] + 0.1295[Z14] + 0.1642[Z25] - 0.0326[Z32] + 0.0683[Z35] - 0.0028[Z44] + 3.0675[T7:A14:T16] + 1.9392[G6:A14] + 1.7280[C12:A14] + 1.3825[T3:T4:A14] + 1.1999[T4:A8] + 0.7989[T4:G6] + 0.1770[T3:G6] + 0.1079[G6:T7]$	0.009011067663210940
PhoB	$\mathrm{logit}[Pr(Y=1)] = -11.1170 + 0.2728[Z2] + 0.0960[Z8] - 0.1738[Z10] + 0.1988[Z12] + 0.2321[Z16] + 1.3089[Z18] - 0.0866[Z19] - 0.4064[Z24] + 0.0438[Z25] + 0.0120[Z28] + 0.3162[Z30] - 0.1671[Z32] + 1.4874[Z35] + 0.3121[Z36] - 0.3152[Z39] - 0.0268[Z40] - 0.1032[Z41] - 0.3318[Z43] - 0.2873[Z44] - 0.3646[Z45] + 1.5717[Z47] + 4.5754[T2:A8:A10] + 2.0487[C1:G3] + 1.8601[A9:T13] + 1.7300[T2:A6] + 1.7094[T2:A9] + 1.5210[A10:T13] + 1.3877[C12:G14] + 1.2372[A8:T13] + 0.9370[T2:A8:A9] + 0.9255[T2:A8:T13] + 0.6432[T2:T13]$	0.001488785883000250
PhoP	$\mathrm{logit}[Pr(Y=1)] = -6.8074 - 0.2106[Z2] + 0.1117[Z5] + 0.0988[Z6] - 0.0041[Z10] - 0.1098[Z11] - 0.1507[Z15] + 0.1143[Z19] + 0.1720[Z20] + 0.2572[Z23] + 0.0750[Z28] + 0.2601[Z30] + 0.3498[Z32] - 0.1107[Z36] - 0.3531[Z47] + 1.4889[T3:A6:A17] + 1.2885[G13:T14:A17] + 1.2404[T3:T5:A17] + 1.0143[A6:T15:A17] + 0.9890[T3:T12:A17] + 0.9344[G13:T14:T15] + 0.8884[T4:T14:T15] + 0.8642[T3:G13:T14] + 0.6504[T16:A17] + 0.5463[T3:T14:T15] + 0.5367[T5:A17] + 0.4256[T3:T4:G13] + 0.3422[T14:T15:A17] + 0.2998[G13:T14] + 0.1774[T3:T14:A17] + 0.1475[T3:T5:T14] + 0.0404[T3:G13:A17]$	0.005753468338614120
PurR	$\mathrm{logit}[Pr(Y=1)] = -14.7961 - 0.0452[Z29] - 0.1313[Z40] + 1.5325[G9:T12:C14] + 1.4537[A6:T11:T16] + 1.4193[A5:A6:C8:C14] + 1.3649[G3:A6:C8:T11] + 1.2509[A5:C8:T16] + 1.1959[A1:G3:T11] + 1.1448[G3:A7:C8] + 1.1395[C8:T12:C14] + 1.1151[A1:G3:C8] + 1.1085[A5:G9:T16] + 1.0362[G3:A7:G9] + 1.0247[A5:T12:C14] + 0.9844[A5:C8:T10] + 0.8830[A6:C8:G9:C14] + 0.8825[G3:A5:C8:T11] + 0.8421[A5:A6:C8:T12] + 0.8329[C8:T11:T16] + 0.8019[A5:T11:T16] + 0.7282[G3:A6:G9:T11] + 0.7124[G3:T11:T16] + 0.6588[C4:G9:C14] + 0.6547[A5:G9:T10] + 0.6096[A6:T12:C14] + 0.5747[G3:A5:A6:C8] + 0.5058[G3:A6:C8:T12] + 0.4998[G3:A5:A6:T11] + 0.4820[G3:A5:C8:G9] + 0.4792[C4:A5:C14] + 0.4768[A6:C8:G9:T12] + 0.4275[G3:A5:G9:T11] + 0.4054[C4:A6:G9] + 0.3607[G3:A5:A6:G9] + 0.3509[C4:A6:C8] + 0.3053[A5:C8:T11:C14] + 0.2779[C4:A5:G9] + 0.2713[C4:A5:C8] + 0.2619[G3:A6:C8:G9] + 0.2581[G3:C8:G9:T11] + 0.2365[A5:G9:T11:C14] + 0.2279[A5:A6:C8:T11] + 0.2261[C4:A6:C14] + 0.1762[C4:G9:T11] + 0.1683[C4:A5:A6] + 0.1561[A6:C8:T11:C14] + 0.1463[G3:G9:T10] + 0.1370[A5:G9:C14] + 0.1343[G3:C8:T10] + 0.1123[C4:A6:T11] + 0.0877[C4:C8:G9] + 0.0866[A6:T10:T11] + 0.0417[A5:C8:G9:T11] + 0.0402[A6:C8:T11:T12] + 0.0247[A6:G9:T11:C14] + 0.0001[A5:A6:G9:T11]$	0.000146174818947755
PutA	$\mathrm{logit}[Pr(Y=1)] = -10.2851 + 2.8316[G3:T4:G6] + 2.7803[G3:G6:A8] + 1.5392[T4:G6:C7] + 1.2763[T5:G6:C7] + 1.1872[T4:T5:G6] + 1.0233[G3:T4:C7] + 0.9719[G3:T5:C7] + 0.9194[G6:C7:A8] + 0.8858[G3:G6:C7] + 0.6927[G3:C7:A8] + 0.6640[G3:T5:G6] + 0.6621[T4:C7:A8] + 0.4106[T5:G6:A8] + 0.3178[T5:C7:A8] + 0.2356[T4:T5:C7] + 0.0483[G3:T4:T5]$	0.000435009701044450
RcsAB	$\mathrm{logit}[Pr(Y=1)] = -6.1764 - 0.2203[Z2] + 0.2327[Z4] + 0.2604[Z7] + 0.7929[Z9] + 0.1358[Z10] + 0.0283[Z21] - 0.5365[Z22] - 0.2285[Z24] + 0.5129[Z26] + 0.0027[Z29] - 0.4159[Z31] - 0.2522[Z32] - 0.0569[Z33] - 0.1585[Z36] - 0.2443[Z44] + 2.7298[G2:A13:A14] + 2.0851[C10:A13] + 1.2081[T5:A13:A14]$	0.009430671047205810

TF	The ELRM	The optimal λ
RcsB	$\text{logit}[Pr(Y=1)] = -6.3607 - 0.0392[Z9] + 0.1126[Z13] - 0.1416[Z25] + 0.3894[Z38] - 0.1194[Z40] - 0.2733[Z42] + 2.3828[A1:G6:A7] + 1.5049[G6:T9] + 1.0731[T3:G6] + 0.8674[G6:A7] + 0.5619[A1:G6:A10] + 0.4581[A1:G6] + 0.3813[T3:A7] + 0.2025[A4:G6] + 0.1969[A1:A7:A10] + 0.0167[A1:A7]$	0.014901900773429300
RelBRelE	$\text{logit}[Pr(Y=1)] = -10.2747 + 10.2939[T3:C5:A6] + 1.9609[A4:C5] + 1.2493[C5:A6] + 1.1228[T3:A6] + 0.9238[C5:A7] + 0.8457[T3:A4] + 0.2672[A4:A6] + 0.1062[T2:A6]$	0.000273601086749336
RhaS	$\text{logit}[Pr(Y=1)] = -5.8935 - 0.0033[Z4] + 0.1063[Z16] - 0.0084[Z19] - 0.2957[Z21] - 0.1421[Z24] + 0.1189[Z32] + 0.0123[Z33] + 0.1652[Z42] - 0.4518[Z47] - 0.2981[Z48] - 0.0514[Z50] - 0.0993[Z51] + 1.7806[T1:C13] + 1.7252[T1:T9] + 1.6917[T1:C10] + 0.8552[T15:T16] + 0.1850[T1:A5]$	0.018738890063109100
Rob	$\text{logit}[Pr(Y=1)] = -6.5243 + 0.1758[Z9] + 0.2425[Z20] + 1.9252[A1:A6:A16] + 1.8661[A1:G4:A16] + 1.6888[A1:C5:A16] + 1.0270[A1:G13:A16] + 0.9928[A1:A16:A17] + 0.9034[A1:A10:A16] + 0.6637[T15:A16] + 0.5587[A1:G3] + 0.1568[C5:A17] + 0.1530[A6:A16] + 0.1276[C5:A16] + 0.0020[G4:A16] + 0.0017[A1:A6]$	0.014027383456660600
RstA	$\text{logit}[Pr(Y=1)] = -10.9381 + 3.5497[G9:T10:T11] + 2.5954[T5:G9:T11] + 2.3843[T10:T11:A14] + 1.4473[T5:T11:C13] + 0.9258[T5:T7:C13] + 0.7591[T7:T10:C13] + 0.7523[T7:T10:A14] + 0.6279[T5:T10:C13] + 0.6238[T5:T10:T11] + 0.5970[T10:T11:A12] + 0.5057[A12:C13] + 0.4034[T7:T10:T11] + 0.3801[T5:T10:A14] + 0.3784[T5:T7:A12] + 0.3527[T5:T7:T11] + 0.2404[T5:T7:A14] + 0.2091[T5:T11:A14] + 0.1677[T5:T10:A12] + 0.1294[C3:T5] + 0.0356[T5:T7:T10] + 0.0326[T7:G9]$	0.000353558946257264
RutR	$\text{logit}[Pr(Y=1)] = -7.9815 + 2.6126[C3:G10:A14] + 2.1159[T8:C12:A14] + 2.0811[C3:T8:C12] + 1.1781[T8:G10] + 0.7605[C3:T8:A14] + 0.6390[C3:C12:A14] + 0.5671[G10:C12] + 0.5098[G10:T11] + 0.4076[C3:C4] + 0.3089[T7:G10]$	0.006288714211936810
SlyA	$\text{logit}[Pr(Y=1)] = -6.4714 + 0.0260[Z7] + 4.0580[A1:G10:A11] + 1.2024[A1:A7:A11] + 0.8513[T2:G10] + 0.7466[A9:G10] + 0.7336[T2:A11] + 0.2456[C8:G10] + 0.2249[A1:G10] + 0.2143[T5:A11] + 0.1950[A1:T5] + 0.1381[G10:A11] + 0.1000[A7:G10]$	0.014574859851042900
SoxS	$\text{logit}[Pr(Y=1)] = -9.1265 - 0.5999[Z3] - 0.0419[Z7] - 0.0094[Z8] + 0.1991[Z10] - 0.3580[Z11] - 0.4212[Z14] + 0.1842[Z15] - 0.1282[Z16] + 0.2130[Z17] - 0.1812[Z18] - 0.1920[Z23] - 0.0551[Z24] - 0.3817[Z27] - 0.6394[Z28] - 0.5576[Z29] - 0.1476[Z30] - 0.0339[Z32] - 0.5928[Z33] - 0.0901[Z34] - 0.8547[Z35] + 0.6088[Z36] + 0.3246[Z42] + 1.0383[Z47] + 0.3903[Z49] - 0.3435[Z52] - 0.6735[Z54] + 3.0861[T2:T3:G14] + 2.7513[T3:T13:G14] + 2.4110[T13:C15] + 2.1524[C15:T18] + 0.9311[T3:G14:T18] + 0.7435[T2:G14:T18] + 0.3901[T2:T3:C15] - 0.3469[T2:G14:C15]$	0.002362604565281920
TorR	$\text{logit}[Pr(Y=1)] = -6.8990 - 0.3680[Z1] - 0.5439[Z2] - 0.1886[Z3] - 0.5872[Z7] - 0.1555[Z8] + 0.6680[Z10] + 0.0006[Z12] - 0.2618[Z17] - 0.0259[Z21] - 0.2572[Z23] - 0.1538[Z24] - 0.1419[Z25] - 0.2041[Z27] + 2.0680[T6:C7] + 1.9035[G4:T5] + 1.6956[G4:T6:T9] + 1.5283[C2:A8] + 0.9224[C2:T3] + 0.8675[C2:T6] + 0.7846[C2:G4:T6] + 0.3160[C7:T9] + 0.2248[C2:T9]$	0.008109805748045720
TrpR	$\text{logit}[Pr(Y=1)] = -6.2106 + 1.6522[C9:G12:T13] + 1.2316[C1:G12:T13] + 0.9156[C1:C9:T13] + 0.6991[T13:A14] + 0.6892[C1:T13:C15] + 0.6319[C1:T5:C9] + 0.4517[A11:G12] + 0.4179[T10:G12] + 0.3647[G12:T13:C15] + 0.3430[C1:T5:C15] + 0.3234[C9:G12:C15] + 0.3075[C9:T10] + 0.2602[T5:C9:T13] + 0.1979[C1:T5:T13] + 0.0654[C1:G12:C15] + 0.0434[T5:G12:C15]$	0.021145626114438100
TyrR	$\text{logit}[Pr(Y=1)] = -16.2427 - 0.0015[Z4] - 1.1091[Z14] - 0.6242[Z15] - 0.2983[Z22] - 0.1910[Z24] + 0.0501[Z30] + 0.1449[Z32] - 0.5493[Z38] + 4.9509[T13:C16:A17] + 2.9334[G1:A15:C16] + 2.6212[G1:A3:C16] + 2.4576[T2:A5:A17] + 1.8746[G1:A15:A17] + 1.6967[G1:A5:C16] + 1.5631[G1:A3:A17] + 1.4916[A15:C16:A17] + 1.4368[T2:A4:A17] + 1.4366[T2:A4:C16] + 1.3848[G1:T2:A4] + 1.2095[G1:A5:A17] + 1.1368[A4:A5:A17] + 1.1013[A4:A5:C16] + 0.9385[G1:T2:C16] + 0.7354[G1:A4:C16:A17] + 0.6445[A3:C16:A17] + 0.5735[T13:A17] + 0.5098[G1:T2:A17] + 0.4973[T2:C16:A17] + 0.4862[A5:C16:A17] + 0.4175[G1:T14:A17] + 0.3644[G1:C16:A17] + 0.1404[G1:T14:C16]$	0.000182639395300399
UlaR	$\text{logit}[Pr(Y=1)] = -11.8618 + 2.2383[C3:A10:A14] + 1.6564[C3:A11:A17] + 1.6323[T2:A11:A17] + 1.5191[T2:A11:A14] + 1.1684[T2:A4:A17] + 1.0139[C3:A4:A17] + 0.9926[C3:A11:A14] + 0.9833[A10:A11:A17] + 0.7706[A4:C13] + 0.6650[T2:A14:A17] + 0.6357[T2:A10:A14] + 0.6114[T2:A4:A14] + 0.5006[T2:A11:T12] + 0.4765[A4:A10:A14] + 0.4084[T2:C13] + 0.3900[C3:A4:A14] + 0.3719[A4:T12:A17] + 0.3701[A4:A10:A17] + 0.3081[C3:A4:A11] + 0.2992[A11:A14:A17] + 0.2033[A4:A11:A14] + 0.1992[A10:T12:A14] + 0.1977[C3:A14:A17] + 0.1572[C3:A11:T12] + 0.1198[A10:C13] + 0.0606[C13:A16] + 0.0562[T2:A4:A11] + 0.0042[T2:T12:A14]$	0.000325178110809082
UxuR	$\text{logit}[Pr(Y=1)] = -5.6366 + 1.5930[T3:A9:A12] + 0.8540[T3:T7:A12] + 0.8528[C11:A12:T15] + 0.7167[T6:T7:A12] + 0.5433[G4:T7] + 0.4004[T6:A12:T15] + 0.3828[T3:A9:C11] + 0.3623[T3:T6:C11] + 0.2692[T6:A9:A12] + 0.2431[T6:C11:A12] + 0.0996[T7:C11:T15] + 0.0831[T3:A12:T15] + 0.0820[T7:A9:T15] + 0.0610[T6:C11:T15] + 0.0604[T3:T6:A12] + 0.0594[T3:C11:T15] + 0.0362[T3:C11:A12] + 0.0230[A9:C11:T15]$	0.030750754087385000
XylR	$\text{logit}[Pr(Y=1)] = -7.7322 + 0.1681[Z3] - 0.1776[Z16] - 0.1493[Z19] - 0.0751[Z21] - 0.1928[Z28] - 0.3532[Z37] - 0.4710[Z48] + 1.8728[A11:G15] + 1.5410[A2:G15] + 1.3929[T13:G15] + 1.2569[A2:A4:A12] + 1.0448[A4:A11] + 0.9201[A2:C8] + 0.8514[A3:A4:A12] + 0.4239[A4:A5:A12] + 0.3534[A2:A12] + 0.2858[A2:A4] + 0.0339[T10:A11]$	0.007264683546198970

Supplementary Table S3. Performance assessment of ELRM and other approaches. We assessed the learning performance of ELRM and three other modeling approaches: PWM,[5] TFFM,[6] DWM.[7] We conducted the ten-fold cross-validation when the positive training size is greater than ten sequences and used the leave-one-out cross-validation for the remaining sets. The same cross-validation procedure was performed for the four approaches. The results from the N-folds were combined and cross-validation AUCs was calculated and shown in this table. In average, the ELRM has significantly lower average AUCs of 0.0172 ± 0.0296 and 0.0180 ± 0.0297 (mean±SD), compared to PWM and TFFM, respectively (Both p-values $< 1e-6$). The ELRM has slightly higher AUCs of 0.0243 ± 0.0733 than the AUC of DWM (p-value = 0.0014).

TF	Positive training set size	PWM	TFFM	DWM	ELRM
Ada	4	0.99	0.91	0.99	0.99
AgaR	11	1.00	0.99	1.00	0.98
AraC	15	0.98	0.81	0.98	0.98
ArcA	75	0.98	0.90	0.98	0.98
ArgP	16	0.99	0.71	0.99	0.96
ArgR	27	0.98	0.97	0.97	0.96
AscG	7	1.00	0.99	1.00	1.00
AsnC	4	1.00	0.99	1.00	1.00
BaeR	4	1.00	1.00	1.00	0.99
CaiF	4	1.00	1.00	1.00	1.00
CpxR	57	0.99	0.86	0.99	0.97
Cra	32	1.00	0.95	1.00	0.96
CRP	236	0.99	0.97	0.99	0.99
CsgD	21	0.98	0.93	0.99	0.97
CysB	10	1.00	0.70	1.00	0.99
CytR	17	0.98	0.91	0.99	0.98
Dan	5	0.99	0.99	0.99	0.98
DcuR	6	0.99	0.99	1.00	0.99
DeoR	7	0.99	0.99	0.99	0.97
DgsA	7	1.00	1.00	1.00	1.00
DnaA	11	1.00	0.99	1.00	0.91
EvgA	7	1.00	1.00	1.00	1.00
ExuR	6	1.00	1.00	1.00	1.00
FadR	14	1.00	0.98	0.99	0.99
FhlA	7	0.99	0.98	0.98	0.99
Fis	211	0.99	0.95	0.99	0.98
FlhDC	16	1.00	0.95	1.00	0.96
FNR	83	0.99	0.97	1.00	0.99
Fur	47	0.98	0.92	0.98	0.96
GadE	5	1.00	1.00	1.00	1.00
GadW	17	0.97	0.89	0.97	0.92
GadX	24	0.98	0.89	0.97	0.96
GalR	12	1.00	0.91	1.00	0.90
GalS	12	1.00	0.99	1.00	1.00
GcvA	4	0.99	0.96	0.99	0.99
GlpR	17	0.98	0.93	1.00	0.99
GntR	9	0.96	0.98	0.95	0.88
HipB	4	1.00	1.00	1.00	1.00
HNS	48	0.98	0.85	0.98	0.98
IclR	10	0.95	0.95	0.94	0.89
IHF	95	0.98	0.90	0.98	0.98
IscR	11	1.00	0.67	1.00	0.99
LeuO	4	1.00	1.00	1.00	1.00
LexA	37	0.97	0.98	0.98	0.95
Lrp	72	0.97	0.86	0.96	0.97
MalT	15	1.00	1.00	1.00	0.98
MarA	22	0.98	0.89	0.98	0.98
MelR	5	1.00	1.00	1.00	0.99
MetJ	22	0.98	0.97	0.98	0.97
MetR	5	0.99	0.98	0.99	0.99
MlrA	4	1.00	1.00	1.00	1.00

TF	Positive training set size	PWM	TFFM	DWM	ELRM
MntR	6	1.00	1.00	1.00	1.00
ModE	7	1.00	0.98	1.00	0.96
MqsA	5	0.99	0.97	0.99	0.96
MqsAMqsR	4	1.00	1.00	1.00	1.00
Nac	12	0.99	0.72	0.99	0.93
NagC	17	0.99	0.94	0.99	0.95
NanR	6	1.00	1.00	1.00	1.00
NarL	91	0.98	0.95	0.98	0.98
NarP	20	1.00	0.97	1.00	0.98
NhaR	6	1.00	0.94	1.00	0.95
NrdR	6	1.00	1.00	1.00	1.00
NsrR	39	1.00	0.98	1.00	1.00
NtrC	17	1.00	1.00	1.00	0.99
OmpR	20	0.99	0.83	0.99	0.98
OxyR	32	1.00	0.93	1.00	0.98
PdhR	10	1.00	0.99	1.00	0.93
PhoB	19	0.98	0.98	0.98	0.96
PhoP	32	0.98	0.92	0.98	0.98
PurR	21	1.00	1.00	1.00	1.00
PutA	5	1.00	1.00	1.00	1.00
RcsAB	6	1.00	0.95	1.00	0.84
RcsB	7	1.00	1.00	1.00	0.99
RelBRelE	4	1.00	1.00	1.00	1.00
RhaS	6	0.97	0.90	0.96	0.95
Rob	10	0.99	0.95	0.99	0.98
RstA	4	1.00	1.00	1.00	1.00
RutR	5	1.00	1.00	1.00	0.99
SlyA	6	0.99	0.99	0.98	0.96
SoxS	24	1.00	0.94	0.99	0.97
TorR	8	0.99	0.97	0.99	0.85
TrpR	6	1.00	1.00	1.00	0.99
TyrR	19	1.00	0.98	1.00	1.00
UlaR	4	1.00	1.00	1.00	1.00
UxuR	4	0.99	0.99	0.99	0.99
XylR	4	1.00	0.99	1.00	0.92

References

1. Agrawal, R., Imieliński, T. & Swami, A. Mining association rules between sets of items in large databases. *SIGMOD Rec.* **22**, 207–216 (1993).
2. Dong, X. *Mining interesting infrequent and frequent itemsets based on minimum correlation strength*, vol. 7002 of *Lecture Notes in Computer Science*, book section 57, 437–443 (Springer Berlin Heidelberg, 2011).
3. Korhonen, J., Martinmaki, P., Pizzi, C., Rastas, P. & Ukkonen, E. MOODS: fast search for position weight matrix matches in DNA sequences. *Bioinformatics* **25**, 3181–2 (2009).
4. Liefooghe, A., Touzet, H. & Varré, J.-S. *Large scale matching for position weight matrices*, vol. 4009 of *Lecture notes in computer science*, book section 36, 401–412 (Springer Berlin Heidelberg, 2006).
5. Stormo, G. D., Schneider, T. D., Gold, L. & Ehrenfeucht, A. Use of the 'Perceptron' algorithm to distinguish translational initiation sites in *E. coli*. *Nucleic Acids Res* **10**, 2997–3011 (1982).
6. Mathelier, A. & Wasserman, W. W. The next generation of transcription factor binding site prediction. *PLoS Comput Biol* **9**, e1003214 (2013).
7. Siddharthan, R. Dinucleotide weight matrices for predicting transcription factor binding sites: generalizing the position weight matrix. *PLoS One* **5**, e9722 (2010).
